# The impact of non-pharmaceutical interventions on COVID-19 epidemic growth in the 37 OECD member states

**DOI:** 10.1007/s10654-021-00766-0

**Published:** 2021-06-10

**Authors:** Francisco Pozo-Martin, Heide Weishaar, Florin Cristea, Johanna Hanefeld, Thurid Bahr, Lars Schaade, Charbel El Bcheraoui

**Affiliations:** 1 Evidence-Based Public Health Unit, Centre for International Health Protection, Robert Koch Institute, Nordufer 20, 13353 Berlin, Germany; 2 Centre for International Health Protection, Robert Koch Institute, Nordufer 20, 13353 Berlin, Germany; 3 Centre for Biological Threats and Special Pathogens, Robert Koch Institute, Nordufer 20, 13353 Berlin, Germany

**Keywords:** COVID-19, OECD, Non-pharmaceutical interventions, Longitudinal analysis, Linear mixed models, Generalized linear mixed models

## Abstract

We estimated the impact of a comprehensive set of non-pharmeceutical interventions on the COVID-19 epidemic growth rate across the 37 member states of the Organisation for Economic Co-operation and Development during the early phase of the COVID-19 pandemic and between October and December 2020. For this task, we conducted a data-driven, longitudinal analysis using a multilevel modelling approach with both maximum likelihood and Bayesian estimation. We found that during the early phase of the epidemic: implementing restrictions on gatherings of more than 100 people, between 11 and 100 people, and 10 people or less was associated with a respective average reduction of 2.58%, 2.78% and 2.81% in the daily growth rate in weekly confirmed cases; requiring closing for some sectors or for all but essential workplaces with an average reduction of 1.51% and 1.78%; requiring closing of some school levels or all school levels with an average reduction of 1.12% or 1.65%; recommending mask wearing with an average reduction of 0.45%, requiring mask wearing country-wide in specific public spaces or in specific geographical areas within the country with an average reduction of 0.44%, requiring mask-wearing country-wide in all public places or all public places where social distancing is not possible with an average reduction of 0.96%; and number of tests per thousand population with an average reduction of 0.02% per unit increase. Between October and December 2020 work closing requirements and testing policy were significant predictors of the epidemic growth rate. These findings provide evidence to support policy decision-making regarding which NPIs to implement to control the spread of the COVID-19 pandemic.

**Supplementary Information:**

The online version contains supplementary material available at 10.1007/s10654-021-00766-0.

## Introduction

The COVID-19 pandemic and its huge negative impact on health, national economies and social cohesion has created an enormous collective challenge for countries around the globe. As of 20 May 2021, 163.7 million cases and more than 3.3 million deaths have been reported worldwide [1]. To reduce the spread of the disease in the absence of widespread vaccination, governments have been relying on social distancing non-pharmaceutical interventions (NPIs) such as restrictions on gatherings, school closure requirements, or stay at home requirements, as well as on more conventional NPIs such as testing and contact tracing.

There is evidence that the implementation of social distancing NPIs have helped to control the spread of the COVID-19 epidemic and its consequences [2–4]. However, stringent applications of these policies have adverse consequences. They place a huge burden on the economy [5], they have equity implications [6, 7] and they can have strong psychological effects on the population [8, 9]. In addition, the public’s willingness to adhere to restrictions (which has been high throughout the first wave of the pandemic [10]) is likely to wane over time, particularly where end dates to these restrictions are uncertain [11]. Considerations like these outline the fact that the choice of type, mix and intensity of NPIs is, while crucial, a difficult policy decision.

The aim of this study was two-fold. First, to explore the association between the intensity and time delay in the implementation of a wide range of NPIs and the time-varying rate of growth of the COVID-19 epidemic during its initial phase in the 37 member states of the Organisation for Economic Co-operation and Development (OECD). Second, to assess whether any effects found in the initial phase were similar at a later stage of the pandemic. The results of this study can help policy makers make decisions about which NPIs to prioritize in their fight against COVID-19.

## Methods

We conducted a data-driven, longitudinal analysis of the association between the NPI response and the epidemic growth in the initial phase of the COVID-19 pandemic in the OECD member states [12] using country-level information from publicly available databases. To assess if any NPI effects were similar in a later stage of the pandemic, we performed an additional analysis with data from the period October 1-December 31, 2020.

### Initial phase of the epidemic

#### Outcome, predictors, control variables, and sources of data

We modelled the time-varying average daily growth rate (wADGR) of the cumulative weekly number of confirmed COVID-19 cases in each country. The wADGR was calculated using the following formula: $wADGR_{t} = \sqrt[7]{{(N_{t} /N_{{t - 1}} )}} - 1$, where $N_{t}$ is the cumulative number of COVID-19 cases at the end of a given week *t* and $N_{t - 1}$ is the cumulative number of COVID-19 cases at the end of the previous week. For more details see Appendix 1 (Online resource). Data for the cumulative number of confirmed cases each week was sourced from the Oxford COVID-19 Government Response Tracker (OxCGRT).

The wADGR was modelled based on measures of (1) the time-varying intensity of individual NPIs, and (2) the time delay in the initial implementation of NPIs. We considered, first, the set of individual interventions included in the stringency index, a composite index developed by OxCGRT [13] of the severity/intensity of the following nine NPIs, most of which are social distancing interventions: school closing requirements, workplace closing requirements, public events cancelling requirements, restrictions on gatherings, public transport restrictions, stay at home requirements, restrictions on internal movement, international travel controls, and public health information campaigns. The stringency index is measured on a continuous scale of increasing severity/intensity between 0 and 100 [13]. Second, we considered two additional interventions: the testing policy and the contact tracing policy. As a potential proxy for the testing policy, we included the total number of tests performed per thousand population in each country from the start of the outbreak until the end of the study period. Finally, we considered one additional NPI: mask-wearing requirements. We obtained and coded data for this interventions from the World Health Organization (WHO) global policy tracker [14] and a study by Leffler et al. [15]. The time-varying intensity of the above NPIs is measured on an ordinal scale of increasing severity/intensity. Time delay of the public health response in each country was measured as the delay (in days) between the date of the first confirmed case of COVID-19 and the date in which the first in-country social distancing intervention was implemented.

The following country-specific control variables were included: baseline number of cases between the start of the epidemic and the implementation of the first in-country social distancing NPI, weekly temperature, sociodemographic index (SDI), gross domestic product (GDP) per capita based on purchasing power parity, percentage of total population living in urban areas, percentage of gross domestic product spent in health, household size, Palma ratio (a measure of income inequality), and democracy index. We collected data on changes in mobility to assess whether this variable had a confounding role in the association between mask wearing requirements and epidemic growth. Appendix 2 (Online resource) provides a description of all data inputs and data sources.

#### Study period

For each country, we retrieved weekly data on the time-varying cumulative number of cases and on the time-varying intensity of the interventions from the date of the first confirmed case of COVID-19 until July 1, 2020. The study period for the statistical analysis of the impact of the NPIs in each country started two weeks after the implementation of the first in-country social distancing NPI. This was done to allow for the remaining NPIs to be subsequently implemented, as they were not all initiated on the same date in each country. The intensity of the NPIs was measured at the beginning of the study period (week zero) and for the following ten weeks. We measured the wADGR with a two-week lag with respect to the implementation of the NPIs to allow for enough time to pass for the NPIs to have an effect on the wADGR. We chose a two-week lag as this is generally considered the maximum incubation period for COVID-19 [16].

#### Statistical analysis

To investigate the impact of NPI intensity and NPI implementation time delay on the wADGR, we used a longitudinal (i.e. growth) multilevel modelling approach. We modelled 11 weeks of repeated measures of the time-varying outcome and regressors for the 37 OECD member states. In total there were 407 data points. We used a multivariable linear mixed model for longitudinal data (mLMM) with the probit transformation of the wADGR (probit_wADGR) as the response variable. This transformation was done to achieve linearity (see Figure A1, Online resource). We assumed that the random effects (intercepts and slopes of the individual country trajectories of the probit_wADGR) were correlated. To identify the regressors for inclusion in the mLMM, we used maximum likelihood estimation with a forward selection procedure as follows:

- First, we fitted a series of univariate linear mixed models with time and individual NPIs as regressors.

- Second, we selected all the NPIs which had shown to be significant regressors in the univariate models.

- Third, we ranked each of these policies in decreasing order, based on the goodness of fit of the univariate models as expressed by the Bayesian Information Criterion (BIC).

- Fourth, we fitted a series of multivariable forward selection linear mixed models with time and adding each NPI sequentially based on its rank from the previous step. If a particular NPI was not a significant predictor it was excluded from the forward selection models.

- Fifth, we introduced implementation time delay and each control variable individually into the forward selection models. If any of these regressors was statistically significant it was included in the final model.

The following model assumptions were verified: normality in the distribution of the within-country residuals; normality in the distribution of the random effect residuals; homoscedasticity in the residuals over time; and lack of collinearity in the regressors.

To assess the robustness of the mLMM, we re-fitted it using Bayesian estimation with minimally informative priors by means of Integrated Nested Laplace Approximation.

The coefficients of the NPIs in the mLMM with probit_wADGR are expressed in terms of the change in the z-score of a standard normal distribution associated with the change in the intensity of each NPI. To facilitate the interpretation of the NPI coefficients, we completed the analysis by fitting a second model to the mLMM regressors. This model was a multivariable beta regression generalized linear mixed model (mGLMM) with a probit link function using wADGR as the response variable. Use of the mGLMM allowed for the estimation of the average marginal effects (AME) of the NPIs on the wADGR. The AME provide a measure, across all observed data, of the average change in the wADGR which results from changes in the level of intensity of each of the NPIs. We verified the following model assumptions for the mGLMM: adequacy of the probit link function, normality in the distribution of the random-effect residuals, no overdispersion (i.e. uniformity in the distribution of the scaled residuals and uniformity in y-direction of the residuals), and no collinearity between the regressors.

Finally, we used the mGLMM to explore interactions between the individual interventions and the time variable (see Appendix 3, Online resource, for the results of this analysis). The R statistical software [17] was used to fit all models. For the linear mixed models, we used the lme4 [18] and INLA [19] R packages, respectively, for the maximum likelihood and the Bayesian model fitting. For the mGLMM, we used the glmmTMB [20] and DHARMa [21] R packages. Appendix 3 (Online resource) describes in more detail the statistical analysis. The R script is provided in Appendix 6 (Online resource).

### October 1-December 31, 2020

To explore if the results from the initial phase were similar at a later stage of the pandemic, we repeated our analysis for the period October 1-December 31, 2020. The reason for selecting this time period is that in the last quarter of 2020, as had occurred during the initial pandemic phase, there was a tightening of the NPI response in most OECD countries. We followed a similar methodological approach as previously described. Data on NPI intensity and weekly cumulative COVID-19 cases was obtained from OxCGRT. We excluded Turkey because the data on cumulative cases for this country was not consistent (between week eight and nine of the study period, the number of total cumulative cases from the start of the epidemic doubled). For the remaining 36 OECD member states, we measured the intensity of the NPIs for 11 weeks starting at the end of the first week of October and the wADGR with a two-week lag with respect to the intensity of the NPIs. As before, we used a multivariable linear mixed model (mLMM2) with maximum likelihood estimation (re-fitted using Bayesian estimation) and, to calculate the AME, a beta regression generalized linear mixed model with a probit link function (mGLMM2). See Appendix 3 (Online resource) for the result of the statistical analysis.

Ethics approval was not required as all data was aggregated at country level and was retrieved from sources publicly available. No funding was received to undertake this study.

## Results

### Initial phase of the epidemic

In the initial phase of the epidemic, from the time of the first case until the end of the study period in each country, 4,282,881 confirmed cases of SARS-COV-2 were reported across the 37 OECD member countries, with the highest number of cases reported from the United States (1,942,472), the United Kingdom (276,504) and Spain (244,109), and the lowest reported from Latvia (1110), Slovenia (1492) and New Zealand (1515).

**Fig. 1:**
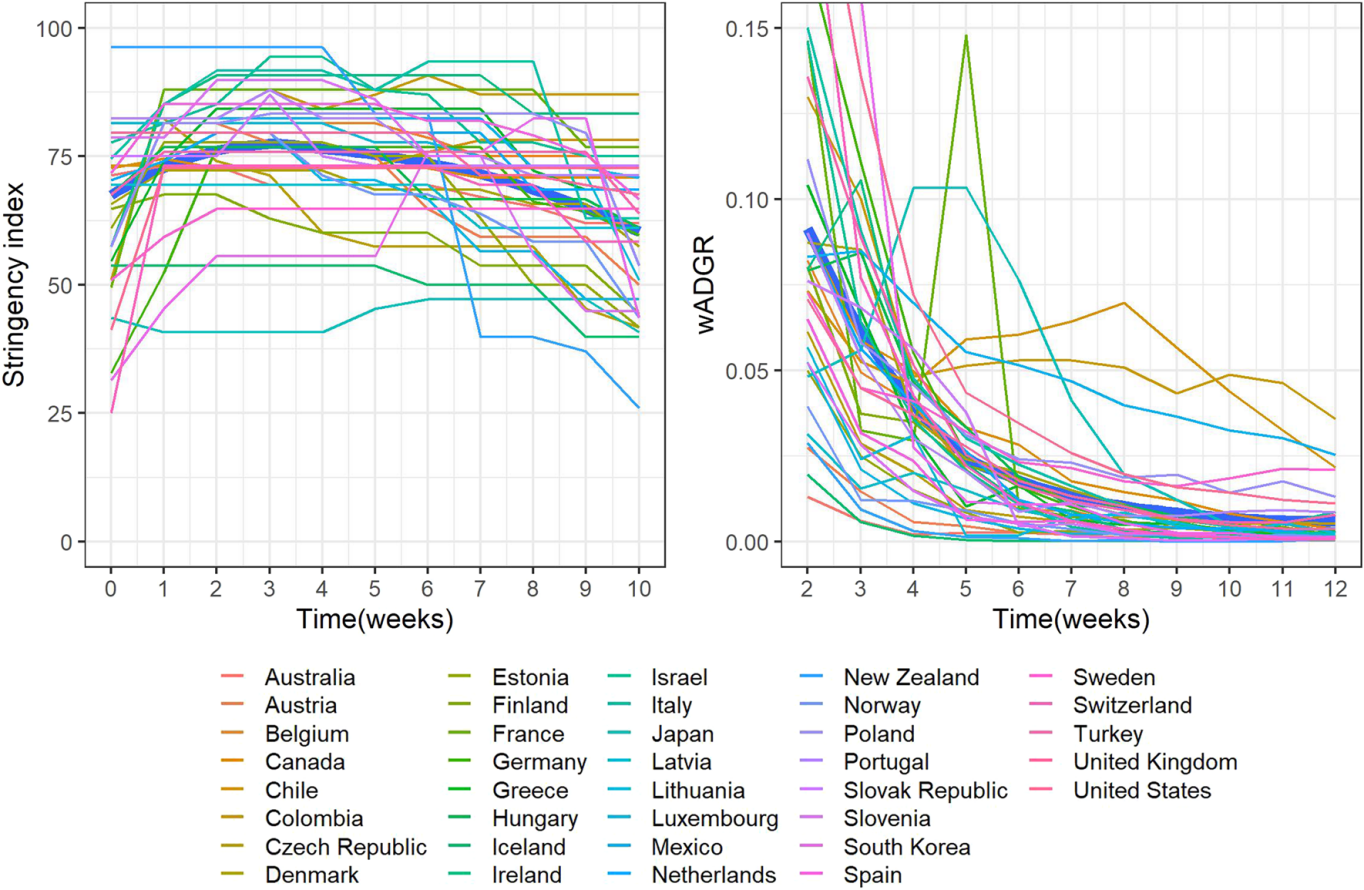
Evolution of the stringency index and the wADGR in the initial phase of the epidemic in 37 OECD member states

Figure 1 presents the change in the stringency index and the wADGR over the 11 weeks studied for each country and overall (thick blue lines, generated using locally estimated scatterplot smoothing).

As observed in Fig. 1, left panel, during the initial phase of the epidemic most countries quickly achieved relatively high levels of NPI intensity as expressed by the stringency index. These levels were in most cases maintained at a constant or near-constant level for several weeks and then progressively relaxed. From the right panel in Fig. 1, the two-week lagged wADGR experienced a maintained exponential decline in most countries, reaching near zero values by the end of the study period. Notable exceptions were Japan (where the wADGR increased in the first four weeks to then begin an exponential decline), Chile, Colombia and Mexico (where the wADGR first declined markedly and then stabilized at a level higher than other countries), and France (where the wADGR in the fifth week experienced a sharp peak).

**Fig. 2:**
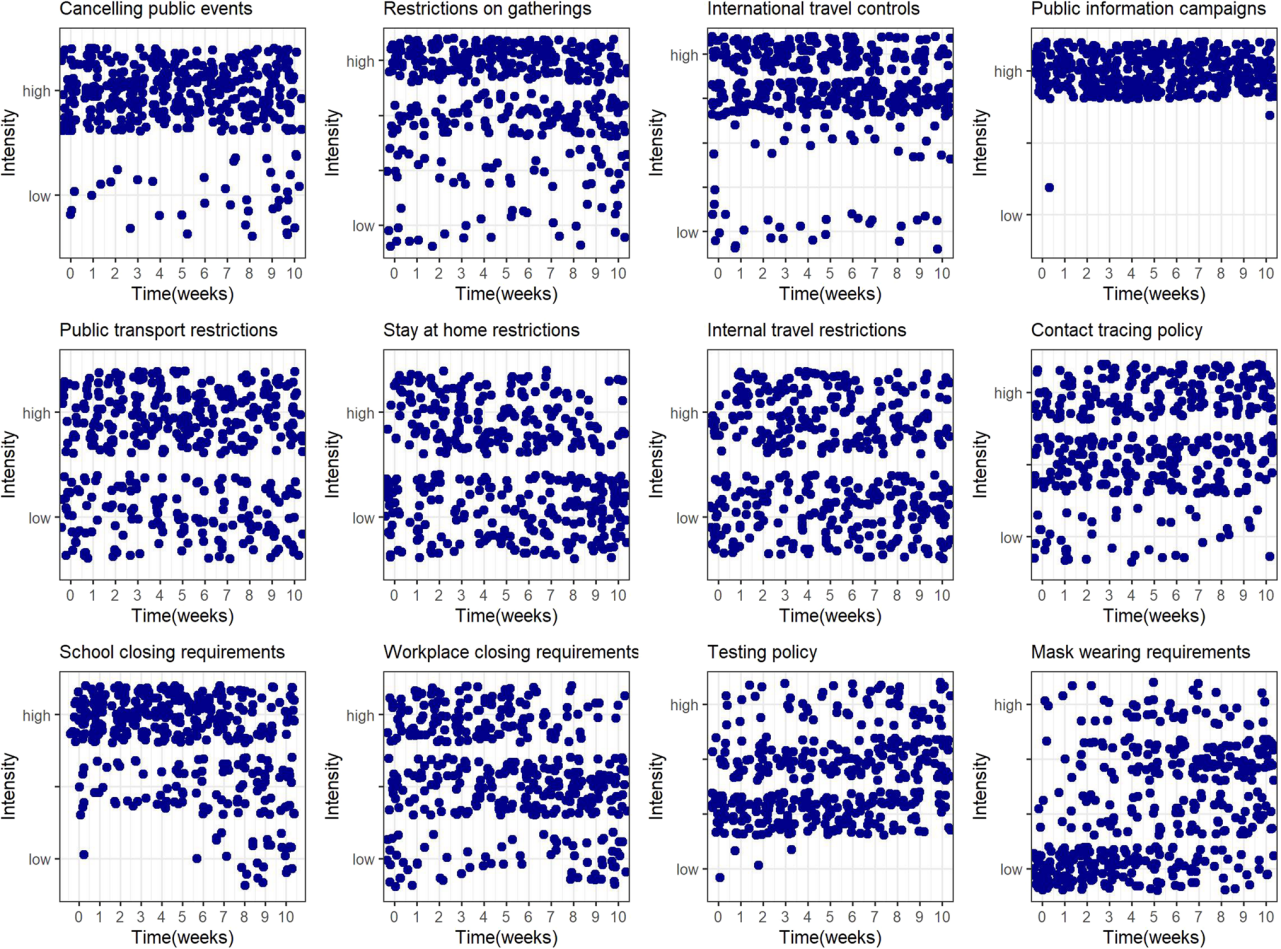
Evolution of the intensity of individual NPIs in the initial phase of the epidemic in 37 OECD member states

Figure 2 presents the change over time in the intensity of the NPIs across countries during the study period of the epidemic’s initial phase (in each graph, each point is the intensity of the corresponding intervention in each country during the relevant week).

Some distinct patterns can be observed in terms of the change in the intensity of implemented NPIs over time. The following NPIs were initially set at relatively high levels of intensity across most countries and were kept at these levels throughout: cancelling of public events, restrictions on gatherings, international travel controls and public information campaigns. Public transport, stay at home, and internal travel restrictions, as well as contact tracing policies were implemented to variable degrees in the different countries but remained relatively constant over time. School closing and workplace closing requirements were initially set in most countries at, respectively, high or moderate to high levels of intensity and kept at those levels until the last 2–3 weeks of the initial phase study period, when they were relaxed. Finally, two NPIs saw their intensity increase across countries: testing policies and, particularly, mask-wearing requirements.

Table 1 shows the results of the multivariable regression models for the initial phase of the pandemic.

**Table 1:** Multivariable model results: initial phase of the pandemic

	mLMM (dependent variable: probit_wADGR)		mGLMM (dependent variable: wADGR)	
Regressors	Maximum likelihood estimation	Bayesian estimation	Restricted maximum likelihood estimation	Average marginal effects (AME) %
	Coefficients (95% CI)	Mean parameters (95% CrI)	Coefficients (95% CI)	
- Intercept	− 0.41 ( − 0.68, − 0.13)	− 0.35 ( − 0.64, − 0.05)	− 0.46 ( − 0.71, − 0.21)	
- Time	− 0.14 ( − 0.15, − 0.12)	− 0.14 ( − 0.19, − 0.08)	− 0.13 ( − 0.15, − 0.11)	− 0.72
- Restrictions on gatherings: gatherings of more than 100 people not permitted	− 0.44 ( − 0.63, − 0.24)	− 0.48 ( − 0.68, − 0.28)	− 0.35 ( − 0.51, − 0.19)	− 2.58
- Restrictions on gatherings: gatherings of between 11 and 100 people not permitted	− 0.66 ( − 0.85, − 0.47)	− 0.70 ( − 0.89, − 0.50)	− 0.39 ( − 0.54, − 0.34)	− 2.78
- Restrictions on gatherings: gatherings of 10 people or less not permitted	− 0.60 ( − 0.78, − 0.41)	− 0.65 ( − 0.83, − 0.46)	− 0.39 ( − 0.42, − 0.27)	− 2.81
- Workplace closing: require closing (or work from home) for some sectors or categories of workers	− 0.18 ( − 0.27, − 0.08)	− 0.17 ( − 0.27, − 0.07)	− 0.24 ( − 0.34, − 0.13)	− 1.51
- Workplace closing: require closing (or work from home) of all-but-essential workplaces (e.g. grocery stores, doctors)	− 0.22 ( − 0.33, − 0.12)	− 0.23 ( − 0.34, − 0.12)	− 0.29 ( − 0.40, − 0.18)	− 1.78
- School closing: require closing of only some levels or categories, e.g. just high school, or just public schools	− 0.10 ( − 0.22, 0.10)	− 0.13 ( − 0.26, 0.06)	− 0.16 ( − 0.30, − 0.02)	− 1.12
- School closing: require closing of all levels	− 0.20 ( − 0.34, − 0.06)	− 0.23 ( − 0.38, − 0.09)	− 0.25 ( − 0.40, − 0.11)	− 1.65
- Mask-wearing: recommended	− 0.04 ( − 0.15, 0.07)	− 0.04 ( − 0.15, 0.08)	− 0.08 ( − 0.18, 0.01)	− 0.45
- Mask-wearing: required in specific public places country-wide or in specific geographical areas within the country	− 0.09 ( − 0.19, 0.00)	− 0.11 ( − 0.20, − 0.01)	− 0.08 ( − 0.15, − 0.005)	− 0.44
- Mask-wearing: required country-wide in all public places or in all public places where social distancing is not possible	− 0.24 ( − 0.37, − 0.12)	− 0.28 ( − 0.21, − 0.14)	− 0.19 ( − 0.32, − 0.07)	− 0.96
- Total number of tests performed per thousand population	− 0.005 ( − 0.008, − 0.002)	− 0.004 ( − 0.008, − 0.001)	− 0.004 ( − 0.007, − 0.001)	− 0.02

*mLMM* multivariable linear mixed model; *mGLMM* multivariable generalized linear mixed model; *CI* Confidence interval; *CrI* Credible interval

The mLMM with maximum likelihood estimation fitted the data well, explaining 92.7% of the total variance in the outcome (conditional R^2^ = 0.927). The fixed effects (time and NPIs) explained 57.8% of the total variance (marginal R^2^ = 0.578) while the random effects (variability between countries) explained 34.9%. In this model (Table 1, first column), we found that variations in restrictions on gatherings, workplace closing requirements, school closing requirements, mask wearing requirements and the total number of tests performed country-wide per thousand population significantly predicted changes in the probit-transformed average daily growth rate of cumulative weekly COVID-19 cases (probit_wADGR). The Bayesian estimation procedure with minimally informative priors produced similar results to the maximum likelihood estimation (Table 1, second column). Neither the time delay in the implementation of the NPIs nor the control variables were significant predictors of changes in the probit_wADGR between countries.

The AME estimated based on the results of the mGLMM (Table 1, fourth column), indicate that restrictions on gatherings had the highest effect of all NPIs in reducing the daily growth rate in cumulative weekly confirmed COVID-19 cases (wADGR). Changes from “no restrictions on gatherings” to, respectively, “gatherings of more than 100 people not permitted”, “gatherings of between 11 and 100 people not permitted”, and “gatherings of 10 people or less not permitted”, were associated with a respective average reduction in the wADGR of 2.58%, 2.78%, and 2.81%. Workplace closing requirements had the second highest effect, followed by school closing requirements. Mask wearing requirements ranked fourth in terms of effect. As tests per thousand population (a proxy for testing policy) is a continuous variable, we did not rank it with respect to the other NPIs which were ordinal variables. Based on the AME, a “dose–response” relationship was apparent for work closing, school closing and mask wearing requirements, but only marginally for restrictions on gatherings. In the best fitting model with interactions between the NPIs and time, we found a significant and positive interaction of mask wearing requirements with time (see Appendix 3, Online resource) with an associated small reduction in the AME across the NPIs.

### October 1-December 31, 2020

During the period October 1-December 31, 2020 there was a general increase in the intensity of the NPI response in most OECD member states to contain the epidemic.

**Fig. 3:**
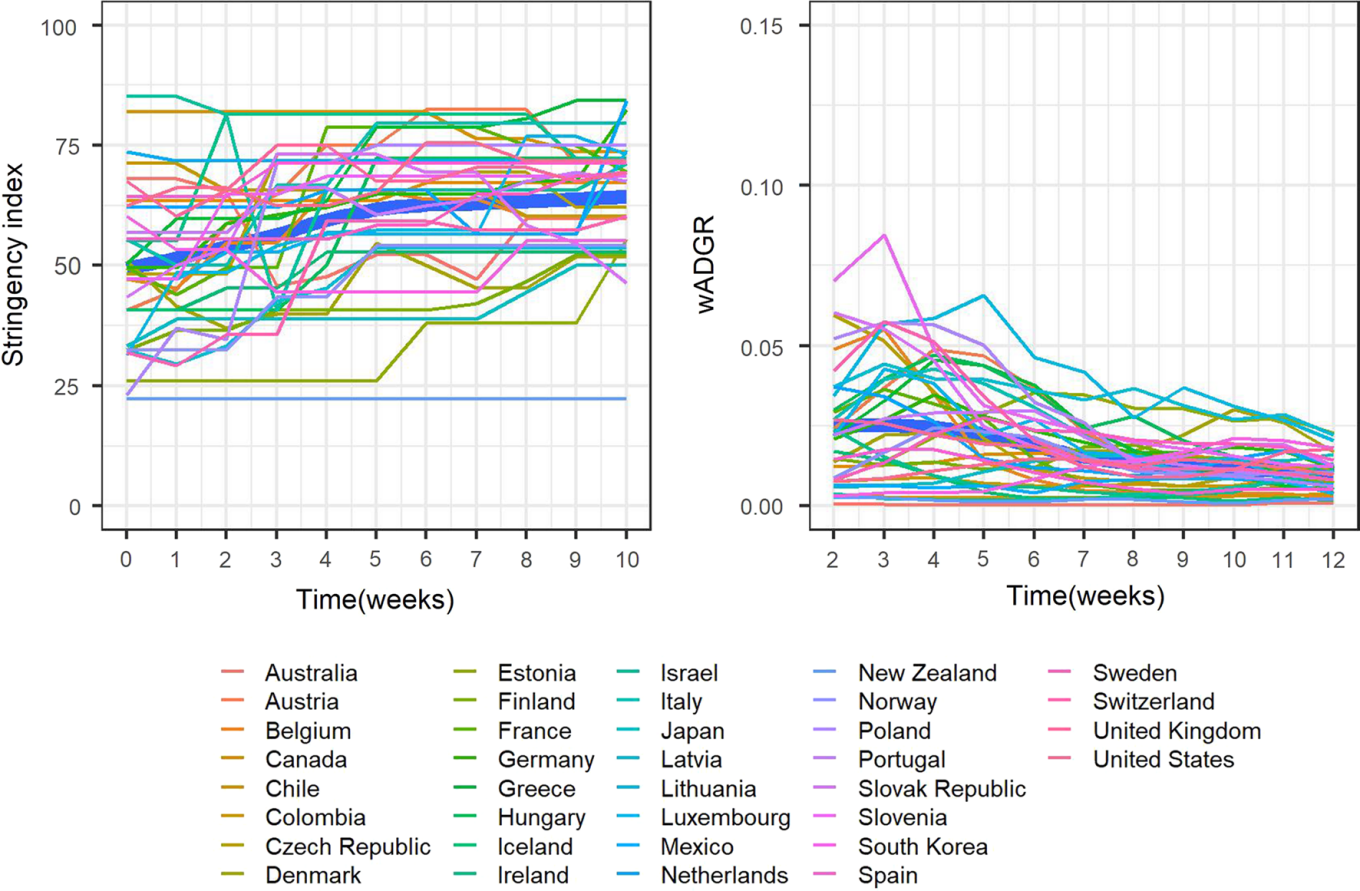
Evolution over time of the stringency index and of the wADGR in OECD states (11 weeks in the period October 1-December 31, 2020)

From Fig. 3, left panel, between October and December 2020 the overall intensity of the NPI response across OECD countries was increased progressively. This is in contrast with the initial phase of the epidemic, in which the policy response was generally swift and aggressive (as evidenced by general high starting levels of stringency, see Fig. 1) and then somewhat relaxed over time. With respect to the wADGR, between October and December 2020 it experienced a slow decline from relatively low starting levels (Fig. 3, right panel). In contrast, as shown in Fig. 1, during the early stages of the epidemic the wADGR experienced an exponential decline from higher starting levels. The individual NPI response between October and December 2020 was different from that of the initial phase of the epidemic (see Appendix 3 for more details).

Table 2 shows the results of the multivariable regression models performed for the period between October and December 2020.

**Table 2:** Multivariable model results: October 1-December 31, 2020

	mLMM2 (dependent variable: probit_wADGR)		mGLMM2 (dependent variable: wADGR)	
Regressors	Maximum likelihood estimation	Bayesian estimation	Restricted maximum likelihood estimation	Average marginal effects (AME) %
	Coefficients (95% CI)	Mean parameters (95% CrI)	Coefficients (95% CI)	
- Intercept	− 1.22 ( − 1.89, − 0.56)	− 0.65 ( − 1.58, 0.27)	− 1.38 ( − 1.90, − 0.74)	
- Time	− 0.03 ( − 0.05, − 0.02)	− 0.03 ( − 0.09, 0.03)	− 0.03 ( − 0.05, − 0.02)	− 0.13
- Workplace closing: require closing (or work from home) for some sectors or categories of workers	− 0.04 ( − 0.09, 0.01)	− 0.03 ( − 0.08, 0.02)	− 0.01 ( − 0.06, 0.05)	− 0.03
- Workplace closing: require closing (or work from home) of all-but-essential workplaces (e.g. grocery stores, doctors)	− 0.21 ( − 0.28, − 0.14)	− 0.20 ( − 0.28, − 0.13	− 0.18 ( − 0.25, − 0.11)	− 0.66
-Testing of anyone showing COVID-19 symptoms	0.17 (0.01, 0.32)	0.19 (0.03, 0.35)	0.28 (0.16, 0.39)	0.89
-open public testing (e.g. “drive through” testing available to asymptomatic people)	0.13 ( − 0.03, 0.30)	0.13 ( − 0.03, 0.30)	0.26 (0.12, 0.40)	0.83
- Percentage of total population living in urban areas	− 0.01 ( − 0.020, − 0.004)	− 0.02 ( − 0.031, − 0.008)	− 0.01 ( − 0.020, − 0.004)	− 0.05

*mLMM2* multivariable linear mixed model 2; *mGLMM2* multivariable generalized linear mixed model 2; *CI* Confidence interval; *CrI* Credible interval

The mLMM2 model fitted the data well, explaining 90% of the total variance in the outcome (conditional R^2^ = 0.902). The fixed effects (time and NPIs) explained 27.5% of the total variance (marginal R^2^ = 0.275) while the random effects (variability between countries) explained 62.7%. Importantly, the results for the initial phase of the epidemic were not similar in the period October-December 2020. We found that the significant predictors of changes in the probit_wADGR were now variations in workplace closing requirements and testing policy. One control variable (percentage of total population living in urban areas) was also a significant predictor. The Bayesian estimation of the mLMM2 produced similar results (Table 2, second column). The AME estimated using mGLMM2 (Table 2, last column) indicate that changes in workplace closing requirements from “no measures or recommend closing (or working from home)” to, respectively, “require closing (or working from home) for some sectors or categories of workers” and “require closing (or working from home) for all but essential workplaces (e.g. grocery stores, doctors)” were associated with an average decrease in the wADGR of 0.03% and 0.66%. Changes from “testing those who both have symptoms and meet specific criteria (e.g. key workers, admitted to hospital, came into contact with a known case, returned from overseas)” to, respectively, “testing anyone showing COVID-19 symptoms” and “open public testing (e.g. “drive-through” testing available to asymptomatic people)” were associated with an average increase in the wADGR of 0.89% and 0.83%. The negative coefficient of the percentage population living in urban areas indicates that, as this percentage increases, the epidemic growth decreases. This result is counterintuitive and may be an artifact: urban areas are typically more crowded than rural areas, hence increasing the probability of contact between any two individuals and as a result increasing the infection rate (and the number of cases).

## Discussion

This cross-country longitudinal study investigated the effect of a comprehensive set of NPIs on the growth of the COVID-19 epidemic during its initial phase in the 37 OECD member states and explored if the results from this initial phase were similar for the period October-December 2020. In the initial phase of the epidemic, (1) restrictions on gatherings, (2) workplace closing requirements, (3) school closing requirements, (4) mask wearing requirements, and (5) the total number of tests performed per thousand population were significant predictors of the average daily growth in cumulative weekly COVID-19 cases, with restrictions on gatherings having the highest effect. For the first four NPIs, higher levels of intensity in the application of the measures tended to be associated with a higher impact on epidemic control, although only marginally for restrictions on gatherings. These results were robust to changes in the model-fitting procedure. The results from the initial phase of the epidemic were not similar in the period October–December 2020. During this period, workplace closing requirements and the testing policy were significant predictors of, respectively, a decrease and an increase in the epidemic growth.

Several data-driven multi-country studies support our findings that restrictions on gatherings were associated with decreases in the COVID-19 epidemic growth in the initial phase of the epidemic [4, 22–25]. Haug et al. [4] found a higher effect of restrictions on small gatherings compared with mass gatherings. Brauner et al. [22] found that limitations on gatherings to 1000 people or less were associated with a 23% reduction in the effective reproduction number R_t_, limitations on gatherings to 100 people or less with a 34% reduction and limitations on gatherings to 10 people or less with a 42% reduction. Liu et al. [23] found that restrictions on gatherings of 1000 people or more were not effective while restrictions on gatherings of 10 people or less were. In contrast, we found only a marginal “dose–response” relationship in the impact of this NPI. Work closing requirements were also shown to be effective in a number of multi-country studies [3, 22, 23, 26, 27] which used data from the first phase of the epidemic. Like us, Brauner et al. [22] and Hunter et al. (preprint) found evidence of a differential effect when different levels of intensity were implemented for this NPI. There is evidence from several studies that school closure requirements have also been effective in the early stages of the epidemic [4, 22, 23, 25, 28–30]. In their analysis with data from 130 countries and territories, Liu et al. [23] found that the effect of school closing on reducing R_t_ was present both when this NPI was implemented at any level of intensity and when it was implemented only at the highest intensity (which is evidence of an effect at different levels of intensity). This is consistent with our finding of a “dose–response” relationship in the effect of school closing requirements. Our findings that mask wearing requirements were a significant predictor of the reduction in epidemic growth in the early stages of the epidemic are supported by other data-driven studies, such as Bo et al. [31] and Chernozhukov [32]. Leffler et al. [15] found that in countries with cultural norms or policies supporting mask wearing, per capita mortality increased less than in other countries. However, there is some debate in the literature regarding the impact of the use of face masks by the public. In a recent meta-analysis of RCTs exploring the use of masks to prevent the transmission of respiratory infections in community settings, Gomez-Ochoa et al. [33] found no effect. More recently, in a multi-disciplinary review of the literature, Howard et al. [34] concluded that the preponderance of the evidence supports the widespread use of face masks by the public. Further research is required to understand the effect of wearing face masks on disease transmission in community settings, including research on the impact of different types of masks and on the impact of adherence to face mask use. With regards to our findings that the number of tests per thousand population were associated with the decrease in the rate of growth of the epidemic in its initial stage, Chaudry et al. [35] found an opposite effect: in their study, testing volume was a significant predictor of the increase in the number of COVID-19 cases per million population in 50 countries. Koh et al. [36] and Islam et al. [24] found that early implementation of lock-down type NPIs have been effective at containing the epidemic. In contrast, we did not find that the delay in the initial NPI response to the epidemic was a predictor of the flattening of the epidemic growth in OECD countries.

Given that most OECD countries implemented strong social distancing measures during the initial phase of the COVID-19 epidemic, it is surprising that stay at home requirements and restrictions on internal movement did not have an impact on epidemic growth during that time. One possible explanation is that, during the 11 weeks studied, these two policies were implemented by OECD countries as recommendations (rather than actual restrictions or bans) much more frequently than was the case for other NPIs such as restrictions on gatherings, work closing and school closing requirements. Similarly, mask wearing requirements had a relatively small absolute effect on the wADGR. This may be partially explained by the fact that the intensity of this NPI increased relatively slowly across the OECD member states over the 11 weeks compared to other NPIs. Two NPIs standard to many health systems that did not impact on the epidemic growth rate in the initial phase of the epidemic were the contact tracing policy and the testing policy. In line with these results, Liu et al. [23] found inconclusive evidence of the effectiveness of these two policies. The lack of effect of contact tracing in the early phase of the epidemic may be explained at least in part by the fact that for the most part of the 11 weeks studied, most OECD countries did not trace the contacts of all confirmed cases. Similarly, a possible reason for the lack of effect of testing policies was that comprehensive testing strategies (i.e. testing of anyone with COVID-19 symptoms or open public testing) were not widespread in the OECD member states until the last few weeks. Our model did, however, identify the volume of testing per unit of population (a proxy for the testing policy) as a significant predictor of epidemic growth.

Our study detected significant variability across countries regarding the impact of the NPI response on the epidemic growth in the early stage of the epidemic, as evidenced by the percentage of the total outcome variance (34.9%) that is unexplained by the fixed effects in the mLMM. Besides differences in the implementation time delay, type and intensity of the measures applied in each country, other factors may have been at play to explain this variability. For example, differences in adherence to the NPIs, variations in the quality of epidemiologic data, or unobserved sociodemographic and health system effects across countries. These and other factors can be investigated further with data from subsequent waves of the epidemic.

In the period October-December 2020, the results were not similar to those of the initial phase. Two NPIs were found to have an effect on the wADGR: work closing requirements (with a very small negative effect) and testing policy (with a positive effect). This effect of the testing policy does not have a straightforward interpretation. On the one hand, higher volume of testing will lead to an increase in the number of COVID-19 cases diagnosed. On the other hand, cases diagnosed will typically be isolated and their contacts traced, tested and quarantined if testing positive, which should lead in the mid-term to fewer cases overall. Perhaps the time lag to that mid-term effect is long enough not to be picked up by the model.

The evidence from data-driven multi-country studies analysing the impact of workplace closing requirements on epidemic growth with data from the latter part of 2020 is not consistent. Sharma et al. (preprint), using data between August 2020 and January 2021, found that business closures were more effective than other NPIs at reducing R_t_. They found that the effect was similar (about a 12% reduction on R_t_) for closure of restaurants, night clubs, retail and close contact services (such as hairdressers) and lower for entertainment venues. Wibbens et al. [28] using data between March and November 2020 (a period which also included the initial phase of the pandemic) found that workplace closing had the strongest impact on the epidemic growth rate out of all NPIs evaluated. In contrast, Ge et al. (preprint) found that in the second wave of the epidemic workplace closures were not effective at mitigating COVID-19 transmission. Out of the three studies above including data from late 2020, only Wibbens et al. [28] analysed the impact of the testing policy and found that its impact on the growth rate, albeit negative, was lower than that of most other policies. We found a positive effect of the testing policy on the epidemic growth rate, possibly due to the increase in cases confirmed through testing.

In the initial phase of the epidemic, shifting from the lowest to the highest levels of intensity of the NPIs had the following effects: shifting from no restrictions on gatherings to restrictions of gatherings of 10 people or less was associated with an average reduction of the wADGR of 2.81%, 57% higher than shifting from no workplace closing requirements to requiring closing of all but essential workplaces, 70% higher than closing all school levels, and about three times as much as requiring country-wide mask wearing in all public places or in all public places where social distancing is not possible. Shifting from no mask requirements to requiring country-wide mask wearing in all public places was associated with an average reduction in the wADGR of 0.96%, twice as much as shifting from no mask requirements to recommending mask wearing. These results should be interpreted with care. This is an ecological study, and as such causality in the associations between the NPIs and epidemic growth cannot be unequivocally inferred. Although the results from the initial phase of the pandemic were not similar for the period between October and December 2020, this does not limit their validity. The dissimilarity in results may be partially explained by the differences in the impact of NPIs between initial and further stages of the epidemic. For example, Sharma et al. (preprint), comparing the first and second waves for seven European countries, found that the effect of NPIs on the reproductive number R_t_ was considerably smaller in the second wave compared with the first. The authors argued that a number of factors played a role in lowering the effect of NPIs, including persistent behavioral change in the population (e.g. avoiding close contact) and the generalized adoption of safety measures (e.g. distancing rules). They added that the effects of NPIs on epidemic control in the early stages of the epidemic were measured relative to the population behavior and safety protocols which were prevalent *before* the epidemic started and hence may not adequately inform policy at later stages once these factors have changed.

Our study has a number of limitations. The analysis reflects national level outcomes and policies based on available data. It does not explore the differential impact of NPIs implemented regionally or locally within countries. The characterization of the intensity of the NPIs using a limited number of ordinal levels (as is done in the OxCCGRT and as we have done with the mask wearing requirements) might mask smaller variations in effect. To detect such variations, databases would have to use interval scales to distinguish NPI intensity. In addition, the compilation and inclusion of data on NPI enforcement and adherence could strengthen the results.

Our study adds to the existing literature by exploring, using a data-driven, longitudinal approach, the impact of NPIs on the COVID-19 epidemic growth in the OECD member states during the initial stage of the epidemic, as well as exploring whether any effects found in the initial phase were similar at a later stage of the pandemic. An important methodological feature of our study is the use of mixed effects longitudinal models to explore the impact of NPIs on the average daily growth rate in weekly confirmed cases. These models use repeated measures to simultaneously explain changes in the growth rate within and across countries. The model presented here was robust, as similar results were obtained using different estimation procedures (maximum likelihood and Bayesian estimation).

## Conclusion

Based on data from 37 OECD member states, this study shows that during the initial phase of the COVID-19 epidemic restrictions on gatherings were most effective at epidemic control. Workplace closing requirements, school closing requirements and mask wearing requirements, as well as the volume of testing per unit of population also successfully reduced the average daily growth rate in the cumulative number of weekly confirmed cases of COVID-19 during this period. In the period October–December 2020, work closing was effective at reducing the epidemic growth rate; the testing policy was positively associated with the growth rate. The use of NPIs for epidemic control is important, even with the current advances in immunization. Insofar as SARS-CoV-2 transmission is active, the virus will mutate and some mutations may increase infectivity, virulence and/or lethality. The currently available vaccines prevent the development of serious forms of COVID-19, but it is not fully known how much they prevent transmission of the virus, and we are a long way from seeing the majority of the world population being vaccinated.

## Supplementary Information

Below is the link to the electronic supplementary material.

Supplementary file1 (XLSX 89 kb)

Supplementary file2 (XLSX 151 kb)

Supplementary file3 (PDF 3963 kb)

## Data Availability

The R code for these models is included in Appendix 6 of the Online resource.

## Data availability

All data used in this analysis is included in this submission.
