# Supplementary material for: The impact of non-pharmaceutical interventions on COVID-19 epidemic growth in the 37 OECD member states
Source: Eur J Epidemiol. 2021 Jun 10;36(6):629–40. doi: 10.1007/s10654-021-00766-0 (PMC8192111; doi:10.1007/s10654-021-00766-0)
Supplement: Supplementary file 3 — Supplementary file3 (PDF 3963 kb) [file 10654_2021_766_MOESM3_ESM.pdf]

EUROPEAN JOURNAL OF EPIDEMIOLOGY

# The impact of non-pharmaceutical interventions on COVID-19 epidemic growth in the 37 OECD member states.

*Francisco Pozo-Martin, research scientist[1], Heide Weishaar, research scientist[1], Florin Cristea, researcher[1], Johanna Hanefeld, head[2], Thurid Bahr, researcher[2], Lars Schaade, head[3], Charbel El Bcheraoui, head[1*]*

*[1]Evidence-Based Public Health Unit, Centre for International Health Protection, Robert Koch Institute. Nordufer 20, 13353 Berlin, Germany*

*[2]Centre for International Health Protection, Robert Koch Institute. Robert Koch Institute. Nordufer 20, 13353 Berlin, Germany*

*[3]Centre for Biological Threats and Special Pathogens, Robert Koch Institute. Nordufer 20, 13353 Berlin, Germany*

*Corresponding author: Charbel El Bcheraoui. E-mail: El-BcheraouiC@rki.de.

## SUPPLEMENTARY INFORMATION

**Appendix 1. Calculation of the average daily growth rate in the cumulative number of weekly cases (wADGR).**

The average daily growth rate in the cumulative number of weekly cases (wADGR) is expressed as: $N_t = (N_{t-1})(1 + wADGR_t)^7$, where $N_t$ is the cumulative number of cases at the end of week *t* and $N_{t-1}$ is the cumulative number of cases at the end of week *t-1*. Solving for wADGR:

$$(N_t/N_{t-1}) = (1 + wADGR_t)^7 \ ;$$

$$1 + wADGR_t = \sqrt[7]{(N_t/N_{t-1})} \ ;$$

$$wADGR_t = \sqrt[7]{(N_t/N_{t-1})} - 1$$

To illustrate with an example, if at the end of the fourth week of the epidemic there are 250 cases ($N_4 = 250$) and at the end of the fifth week of the epidemic there are 300 cases ($N_5 = 300$):

$$wADGR_5 = \sqrt[7]{(N_5/N_4)} - 1;$$

$$wADGR_5 = \sqrt[7]{(300/250)} - 1 = 0.026 = 2.6\%$$

The average daily growth rate in the cumulative number of cases in week 5 is 2.6%.

**Appendix 2. Data collection, data inputs, and data sources.**

Table A1 provides a description of the data inputs and their sources. All data was collected via searches in the World Wide Web. The values of some categorical variables were recoded to balance the number of observations between levels (see Table 1). For the mask wearing requirements, we combined country-specific values from two sources – a study by Leffler et al [1] and the WHO COVID-19 Government Response Tracker [2] and coded them ourselves according to three levels: No requirements/ Mask wearing in public recommended / Mask wearing in public required in some public places or in some geographical areas within the country / Mask wearing in public required in all public places and in all geographical areas within the country.

Table A1. Data inputs and data sources

| **Variable** | **Value levels** | **Value levels if recoded** | **Source and period of data collection** |
|---|---|---|---|
| Cumulative number of confirmed cases of COVID-19 | Not applicable | Not recoded | Oxford COVID-19 Government Response Tracker: https:// www.bsg.ox.ac.uk/ research/ research-projects/ coronavirus-government-response-tracker<br><br>January 1, 2020 – December 31, 2020 |
| Stringency index | 0-100 | Not recoded | |
| School closing requirements | 0 - No measures<br>1 - recommend closing<br>2 - Require closing (only some levels or categories, e.g. just high school, or just public schools)<br>3 - Require closing all levels | 0 - No measures or recommend closing<br>2 - Require closing (only some levels or categories, e.g. just high school, or just public schools)<br>3 - Require closing all levels | |
| Workplace closing requirements | 0 - No measures<br>1 - recommend closing (or work from home)<br>2 - require closing (or work from home) for some sectors or categories of workers<br>3 - require closing (or work from home) all-but-essential workplaces (e.g. grocery stores, doctors) | 0 - No measures or recommend closing (or work from home)<br>2 - require closing (or work from home) for some sectors or categories of workers<br>3 - require closing (or work from home) all-but-essential workplaces (e.g. grocery stores, doctors) | |
| Public events cancelling requirements | 0 - No measures<br>1 - Recommend cancelling<br>2 - Require cancelling | 0 – No measures or recommend cancelling<br>2 – Require cancelling | |
| Restrictions on gatherings | 0 - No restrictions<br>1 - Restrictions on very large gatherings (the limit is above 1000 people)<br>2 - Restrictions on gatherings between 101-1000 people<br>3 - Restrictions on gatherings between 11-100 people<br>4 - Restrictions on gatherings of 10 people or less | 0 - No restrictions<br>1 - Restrictions on gatherings of more than 100 people<br>2 - Restrictions on gatherings of between 11 and 100 people<br>3 – Restrictions on gatherings of 10 people or less | |
| Public transport restrictions | 0 - No measures<br>1 - Recommend closing (or significantly reduce volume/ route/ means of transport available)<br>2 - Require closing (or prohibit most citizens from using it) | 0 – No measures<br>1 – Recommend closing (or significantly reduce volume/ route/ means of transport available) or require closing (or prohibit most citizens from using it) | |
| Stay at home requirements | 0 - No measures<br>1 - recommend not leaving house<br>2 - require not leaving house with exceptions for daily exercise, grocery shopping, and 'essential' trips<br>3 - Require not leaving house with minimal exceptions (e.g. allowed to leave only once a week, or only one person can leave at a time, etc.) | 0 – No measures or recommend not leaving house<br>1 - require not leaving house with exceptions for daily exercise, grocery shopping, and 'essential' trips or require not leaving house with minimal exceptions (e.g. allowed to leave only once a week, or only one person can leave at a time, etc.) | |

Table A1. Data inputs and data sources (cont.).

| Variable | Value levels | Value levels if recoded | Source and period of data collection |
|---|---|---|---|
| Restrictions on internal movement | 0 - No measures<br>1 - Recommend not to travel between regions/ cities<br>2 – internal movement restrictions in place | 0 – No measures or recommend not to travel between regions/ cities<br>2 – internal movement restrictions in place | Oxford COVID-19 Government Response Tracker: https:// www.bsg.ox.ac.uk/ research/ research-projects/ coronavirus-government-response-tracker<br><br>January 1, 2020 –<br>December 31, 2020 |
| International travel controls | 0 - No measures<br>1 - Screening<br>2 - Quarantine arrivals from high-risk<br>regions<br>3 - Ban on arrivals from some regions<br>4 - Ban on all regions or total border<br>closure | Not recoded | |
| Public health information campaigns | 0 -No COVID-19 public information campaign<br>1 - public officials urging caution about<br>COVID-19<br>2 - coordinated public information campaign<br>(e.g. across traditional and social media) | Not recoded | |
| Testing policy | 0 – No testing policy<br>1 – Only those who both (a) have symptoms AND (b) meet specific criteria (e.g. key workers, admitted to hospital, came into contact with a known case, returned from overseas)<br>2 – testing of anyone showing COVID-19 symptoms<br>3 – open public testing (e.g. “drive through” testing available to asymptomatic people) | Not recoded | |
| Contact tracing policy | 0 - No contact tracing<br>1 - Limited contact tracing - not done for all cases<br>2 - Comprehensive contact tracing - done for all identified cases | Not recoded | |
| Total number of SARS-COV-2 tests per thousand population | Not applicable | Not recoded | Source for total number of tests - Our World in Data:<br>https://ourworldindata.org/<br><br>January 1, 2020 –<br>July 1, 2020<br><br>Source for population estimations - United Nations Population Division:<br><br>https://population.un.org/<br><br>2020 |
| Mask wearing requirements | 0 - No requirements<br>1 – Mask wearing recommended<br>2 – Mask wearing: required in specific public places country-wide or in specific geographical areas within the country<br>3. Mask wearing: required country-wide in all public places or in all public places where social distancing is not possible country-wide | Not recoded | Leffler (ref)<br>WHO:<br>https://covid19.who.int/<br><br>January 1, 2020-<br>December 31, 2020 |

Table A1. Data inputs and data sources (cont.)

| Variable | Value ranges/ levels | Value ranges/levels if recoded | Source and period of data collection |
|---|---|---|---|
| Baseline number of cumulative confirmed cases | Not Applicable | Not recoded | Oxford COVID-19 Government Response Tracker: https:// www.bsg.ox.ac.uk/ research/ research-projects/ coronavirus-government-response-tracker |
| Weekly temperature | Not applicable | Not recoded | National Oceanic and Atmospheric Administration, Physical Sciences Laboratory: https://psl.noaa.gov/data/composites/day/<br><br>2020 |
| GDP per capita purchasing power parity | Not applicable | Not recoded | OECD: https://stats.oecd.org/<br><br>2019 |
| Socio-demographic Index | 0-1 | Not recoded | Institute of Health Metrics and Evaluation Global Burden of Disease: http://ghdx.healthdata.org/<br><br>2017 |
| % of total population living in urban areas | 0%-100% | Not recoded | World Bank: https://databank.worldbank.org/<br><br>2019 |
| % GDP spent in health | 0%-100% | Not recoded | OECD: https://data.oecd.org/<br><br>2017-2018 |
| Average household size | Not applicable | Not recoded | United Nations Population Division: https://population.un.org/<br><br>2019 |
| Palma ratio | Not applicable | Not recoded | OECD: https://data.oecd.org/<br><br>2015-2019 |
| Democracy index | Not applicable | Not recoded | The Economist: https://www.economist.com/graphic-detail/2021/02/02/global-democracy-has-a-very-bad-year<br><br>2020 |
| Mobility composite | Not applicable | Not recoded | Institute of Health Metrics and Evaluation www.healthdata.org/covid/data-downloads<br><br>2020 |

**Appendix 3. Statistical analysis**.

**3.1. Initial phase of the COVID-19 epidemic**

**3.1.1. Multivariable linear mixed model (mLMM)**

**Outcome and overall approach to model fitting.**

We performed a probit transformation of the ADGR values (probit_wADGR). We then fitted a series of OLS-lines to the probit_ADGR trajectories (see Figure A1). From Figure A1, it was appropriate to use a growth model linear in time.

Figure A1. Probit_ADGR trajectories and fitted OLS lines.

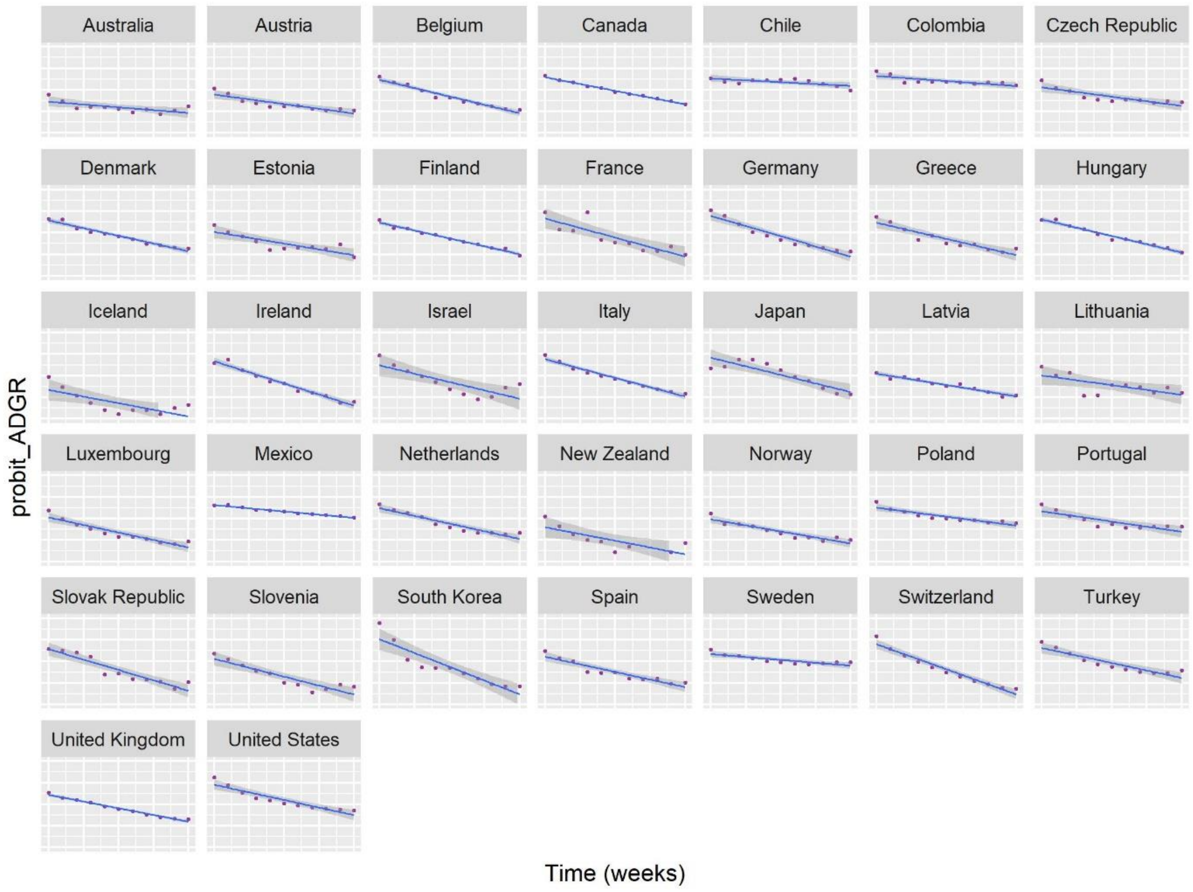


**Model fitting.**

All models were fitted using Maximum Likelihood (ML) estimation with the Nelder-Mead optimiser. Statistical significance was set at p=0.05. In addition, the final model was also fitted using Bayesian Estimation with minimally informative priors using Integrated Nested Laplace Estimation.

The first fitted model was an unconditional growth model with only time as an independent variable (see Table 2).

Table A2. Model 1 results.

| Terms | Coefficients (SE) | p-values | BIC<br>Conditional $R^2$ | Variance (intercept)<br>Variance (slope)<br>Variance (residuals) |
|---|---|---|---|---|
| -Intercept<br>-Time | -1.62 (0.064)<br>-0.13 (0.008) | 0<br>0 | 150.6<br>0.869 | 0.137<br>0.002<br>0.05 |

**Identifying the mLMM: forward selection.**

To identify the regressors for inclusion in the mLMM, we used maximum likelihood estimation with a forward selection procedure as follows:

- First, we fitted a series of univariate linear mixed models with time and individual NPIs as regressors

- Second, we selected all the NPIs which had shown to be significant regressors in the univariate models

- Third, we ranked each of these policies in decreasing order, based on the goodness of fit of the univariate models as expressed by the Bayesian Information Criterion (BIC)

- Fourth, we fitted a series of multivariable forward selection linear mixed models with time and adding each NPI sequentially based on its rank from the previous step. If a particular NPI was not a significant predictor it was excluded from the forward selection models

- Fifth, we introduced implementation time delay and each control variable individually into the forward selection models. If any of these regressors was statistically significant it was included in the final model.

Table A3. Univariate model results and sequence in which terms were entered into the forward selection multivariable linear mixed model.

| Model (rank) | Terms | Coefficients (SE) | p-values | BIC | Variance (intercept)<br>Variance (slope)<br>Variance (residuals) |
|---|---|---|---|---|---|
| Model 2<br>(1) | -Intercept<br>-Time<br>- Restrictions on gatherings (more than 100 people)<br>- Restrictions on gatherings (between 11-100 people)<br>- Restrictions on gatherings (10 people or less) | -0.98 (0.10)<br>-0.13 (0.008)<br>-0.35 (0.10)<br>-0.71 (0.10)<br>-0.72 (0.10) | 0<br>0<br>0<br>0<br>0 | 94.1 | 0.10<br>0.002<br>0.04 |

Table A3. Univariate model results and sequence in which terms were entered into the forward selection multivariable linear mixed model (cont.)

| Model (rank) | Terms | Coefficients (SE) | p-values | BIC | Variance (intercept)<br>Variance (slope)<br>Variance (residuals) |
|---|---|---|---|---|---|
| Model 3<br>(2) | -Intercept<br>-Time<br>-Workplace closing (require closing or work from home for some sectors or categories of workers)<br>-Workplace closing (require closing or work from home all-but-essential workplaces e.g. grocery stores, doctors) | -1.33 (0.07)<br>-0.14 (0.008)<br>-0.28 (0.05)<br><br>-0.34 (0.05) | 0<br>0<br>0<br><br>0 | 123.8 | 0.11<br>0.002<br>0.044 |
| Model 4<br>(3) | -Intercept<br>-Time<br>-Stay at home requirements (require not leaving house with exceptions for daily exercise, grocery shopping, and 'essential' trips or require not leaving house with minimal exceptions e.g. allowed to leave only once a week, or only one person can leave at a time, etc.) | -1.52 (0.069)<br>-0.14 (0.008)<br>-0.15 (0.038) | 0<br>0<br>0 | 142.9 | 0.14<br>0.002<br>0.047 |
| Model 5<br>(4) | -Intercept<br>-Time<br>- School closing (only some levels or categories, e.g. just high school, or just public schools)<br>- School closing (require closing all levels) | -1.30 (0.10)<br>-0.14 (0.009)<br>-0.17 (0.07)<br><br>-0.32 (0.08) | 0<br>0<br>0.02<br><br>0 | 144.9 | 0.14<br>0.002<br>0.045 |
| Model 6<br>(5) | -Intercept<br>-Time<br>-Public events cancelling requirements (require cancelling) | -1.38 (0.09)<br>-0.14 (0.008)<br>-0.24 (0.07) | 0<br>0<br>0 | 146.1 | 0.13<br>0.002<br>0.048 |
| Model 7<br>(6) | -Intercept<br>-Time<br>-Restrictions on internal movement (internal movement restrictions in place) | -1.54 (0.07)<br>-0.13 (0.008)<br>-0.13 (0.04) | 0<br>0<br>0.002 | 147.2 | 0.14<br>0.002<br>0.046 |
| Model 8<br>(7) | -Intercept<br>-Time<br>-Mask wearing requirements (mask wearing recommended)<br>-Mask wearing requirements (mask wearing required in specific public spaces country-wide or in specific geographical areas)<br>-Mask wearing requirements (mask wearing required country-wide in all public places or in all public places where social distancing is not possible) | -1.58 (0.07)<br>-0.13 (0.009)<br>-0.06 (0.06)<br><br>-0.05 (0.05)<br><br><br>-0.32 (0.07) | 0<br>0<br>0.38<br><br>0.31<br><br><br>0 | 149.2 | 0.14<br>0.002<br>0.046 |

Table A3. Individual policy growth model results and sequence in which terms were entered into the forward selection growth model (cont.)

| **Model (rank)** | **Terms** | **Coefficients (SE)** | **p-values** | **BIC** | **Variance (intercept)<br>Variance (slope)<br>Variance (residuals)** |
|---|---|---|---|---|---|
| Model 9<br>(8) | -Intercept<br>-Time<br>-Public transport restrictions (recommend closing or significantly reduce volume/ route/ means of transport available or require closing or prohibit most citizens from using it) | -1.53 (0.07)<br>-0.13 (0.009)<br>-0.14 (0.06) | 0<br>0<br>0.01 | 150.6 | 0.14<br>0.002<br>0.048 |
| Model 10<br>(9) | -Intercept<br>-Time<br>-International travel controls (screening)<br>-International travel controls (quarantine arrivals from high-risk regions)<br>-International travel controls (ban on arrivals from some regions)<br>-International travel controls (ban on all regions or total border closure) | -1.26 (0.15)<br>-0.13 (0.008)<br>0.04 (0.23)<br>-0.15 (0.15)<br>-0.36 (0.14)<br>-0.44 (0.15) | 0<br>0<br>0.85<br>0.31<br>0.01<br>0.003 | 156.9 | 0.14<br>0.002<br>0.046 |
| Model 11<br>(10) | -Intercept<br>-Time<br>- public officials urging caution about COVID-19<br>-Public information campaigns (coordinated public information campaign e.g. across traditional and social media) | -1.11 (0.26)<br>-0.13 (0.008)<br>-0.33 (0.36)<br>-0.51 (0.26 | 0<br>0<br>0.36<br>0.049 | 158.4 | 0.13<br>0.002<br>0.048 |

Table A4 below shows the multivariate linear mixed model (mLMM) resulting from the forward selection. Results are presented for both the maximum likelihood and Bayesian estimation.

Table A4. mLMM: maximum likelihood and Bayesian estimations

| Model | Terms | ML coefficients (SE) | p-values | BIC | Variance (intercept) Variance (slope) Variance (residuals) | Mean coefficients from Bayesian estimation |
|---|---|---|---|---|---|---|
| Model 12 | -Intercept<br>-Time | -0.41 (0.14)<br>-0.14 (0.009) | 0.003<br>0 | 87.1 | 0.09<br>0.002<br>0.034 | -0.35<br>-0.14 |
| | - Restrictions on gatherings (more than 100 people) | -0.44 (0.098) | 0 | | | -0.48 |
| | - Restrictions on gatherings (between 11-100 people) | -0.66 (0.094) | 0 | | | -0.70 |
| | - Restrictions on gatherings (10 people or less) | -0.60 (0.091) | 0 | | | -0.65 |
| | -Mask wearing requirements (mask wearing recommended) | -0.04 (0.056) | 0.43 | | | -0.04 |
| | -Mask wearing requirements (mask wearing required in specific public spaces country-wide or in specific geographical areas) | -0.09 (0.047) | 0.048 | | | -0.11 |
| | -Mask wearing requirements (mask wearing required country-wide in all public places or in all public places where social distancing is not possible) | -0.24 (0.064) | 0 | | | -0.28 |
| | -Workplace closing (require closing or work from home for some sectors or categories of workers) | -0.18 (0.048) | 0 | | | -0.17 |
| | -Workplace closing (require closing or work from home all-but-essential workplaces e.g. grocery stores, doctors) | -0.22 (0.054) | 0 | | | -0.23 |
| | - School closing (only some levels or categories, e.g. just high school, or just public schools) | -0.10 (0.064) | 0.12 | | | -0.13 |
| | - School closing (require closing all levels) | -0.20 (0.072) | 0.005 | | | -0.23 |
| | -Total number of tests per thousand population | -0.005 (0.001) | 0.006 | | | -0.004 |

In the mLMM, variations in the following regressors were significant predictors of changes in the probit_ADGR: 1) restrictions on gatherings, 2) mask wearing requirements, 3) workplace closing requirements, 4) school closing requirements, and 5) total number of tests performed per thousand population during the study period. The maximum likelihood and Bayesian estimation give very similar results.

**Testing the assumptions of the mLMM.**

In the sub-sections below we present our assessment of the model assumptions, including: Normality in the residuals, heteroscedasticity in the residuals, and lack of strong collinearity in the regressors.

*Normality in the distribution of the residuals*

Figure A2 presents qqplots for, respectively, the level-1 residuals, i.e. the residuals across all countries and all occasions of measurement (top graph) and the level-2 residuals, i.e. the residuals in the intercepts and slopes across countries (bottom graph), i.e. in the between-country intercept and slope residuals. From Figure A2, the distribution of these residuals can be considered approximately Normal.

Figure A2. Level-1 residuals and Level-2 residuals for the mLMM: qqplots

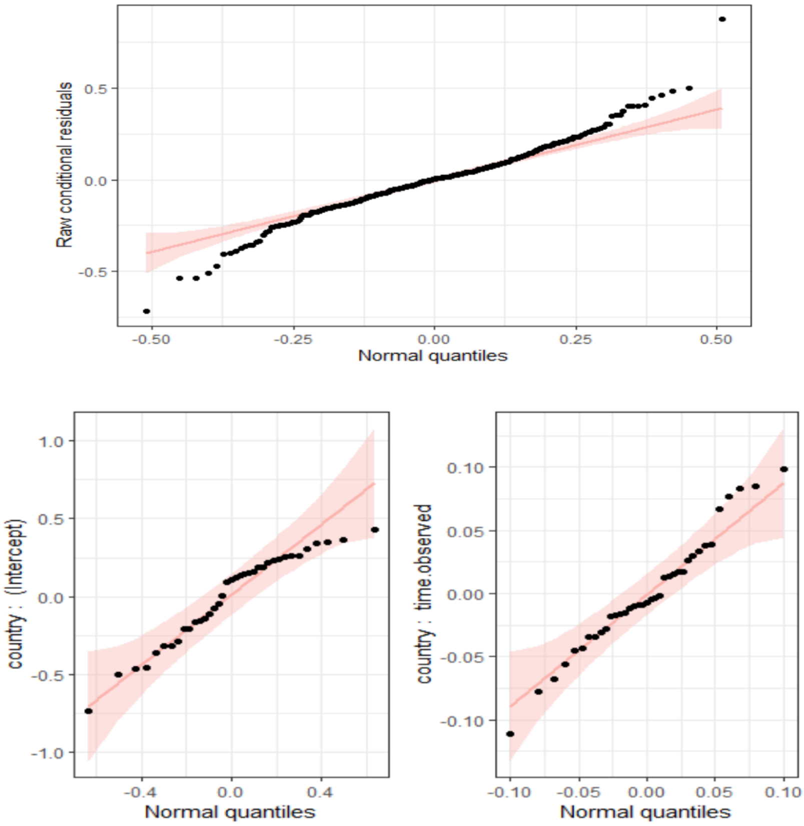

*Homoscedasticity in the distribution of the residuals*

Figure A3 (top graph) maps the studentized level-1 residuals against the final model's fitted values, showing no patterns and an approximately constant variance of these residuals around zero across the predicted values for the outcome. Figure A3 (bottom graph) plots the distribution of these residuals at different time points, showing approximately constant variance across time.

Figure A3. Level-1 residuals for the mLMM: homoscedasticity

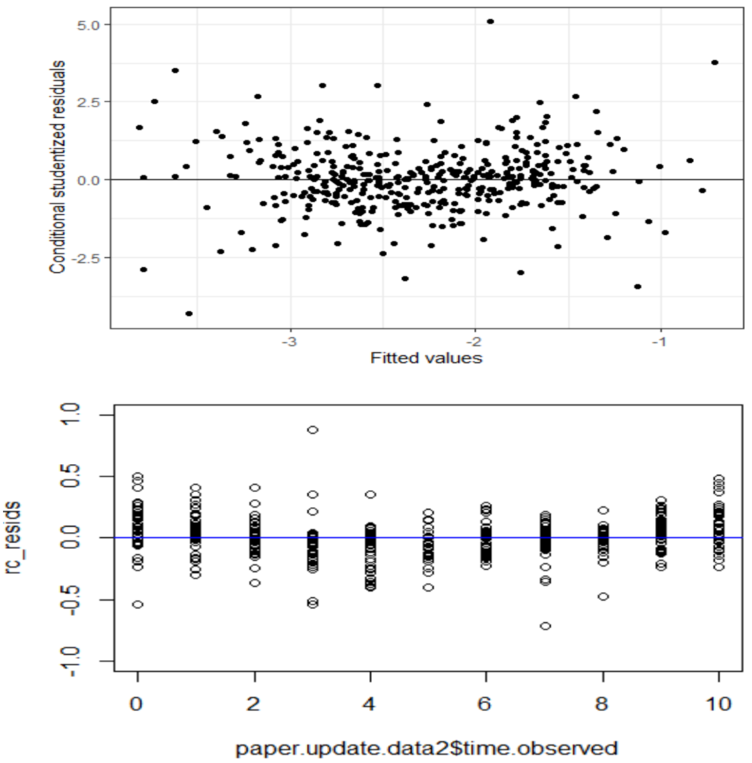


*Lack of strong collinearity between regressors*

Table A5 shows the variance inflation factor (VIF) for each of the regressors in the mLMM. Values close to 1 indicate no collinearity.

Table A5. Variance inflation factor (VIF) for the mLMM regressors

| **Regressors** | **VIF** |
|---|---|
| Time | 1.10 |
| Restrictions on gatherings | 1.07 |
| Mask wearing requirements | 1.04 |
| Workplace closing requirements | 1.09 |
| School closing requirements | 1.07 |
| Tests per thousand population | 1.03 |

While mobility (an independent predictor of the probit_ADGR) can be considered an intermediate variable, i.e.occurring in the causal pathway between most NPIs and the epidemic growth rate and hence should not be included as an explanatory variable in the mLMM, it may be a confounding variable in the association between mask wearing and epidemic growth. In a sensibility analysis, we assessed whether the effect of mask wearing was maintained when mobility was included in the model (see Table A6).

Table A6. Univariate model: mask wearing requirements with mobility changes.

| Model | Terms | Coefficients (SE) | p-values |
|---|---|---|---|
| Sensibility analysis | -Intercept | -0.30 (0.14) | 0.03 |
| | -Time | -0.15 (0.01) | 0 |
| | - Restrictions on gatherings (more than 100 people) | -0.36 (0.10) | 0 |
| | - Restrictions on gatherings (between 11-100 people) | -0.59 (0.09) | 0 |
| | - Restrictions on gatherings (10 people or less) | -0.50 (0.09) | 0 |
| | -Mask wearing requirements (mask wearing recommended) | -0.03 (0.05) | 0.5 |
| | -Mask wearing requirements (mask wearing required in specific public spaces country-wide or in specific geographical areas) | -0.09 (0.09) | 0.04 |
| | -Mask wearing requirements (mask wearing required country-wide in all public places or in all public places where social distancing is not possible) | -0.25 (0.06) | 0 |
| | -Workplace closing (require closing or work from home for some sectors or categories of workers) | -0.06 (0.05) | 0.21 |
| | -Workplace closing (require closing or work from home all-but-essential workplaces e.g. grocery stores, doctors) | -0.07 (0.06) | 0.26 |
| | - School closing (only some levels or categories, e.g. just high school, or just public schools) | -0.07 (0.06) | 0.24 |
| | - School closing (require closing all levels) | -0.12 (0.07) | 0.08 |
| | - Mobility | -0.01 (0.001) | 0 |
| | -Total number of tests per thousand population | -0.004 (0.002) | 0.01 |

From Table A6, mobility picks up the effect of other NPIs but not of mask wearing requirements, which was still present.

### 3.1.2. Multivariable generalised linear mixed model (mGLMM)

We fitted a multivariable beta regression generalized linear mixed model (mGLMM) with a probit link function using the average daily rate of growth in the cumulative weekly cases (wADGR) as the response variable. We used restricted maximum likelihood estimation to fit the model. The mGLMM allowed for the estimation of the average marginal effects (AME) of the NPIs on the wADGR. The AME provide a measure, across all observed data, of the average change in the wADGR which results from changes in the level of intensity of each of the NPIs. The best fitting mGLMM was adjusted by modelling the model dispersion with workplace closing requirements as a regressor. Table A7 presents the model results.

<u>Table A7. mGLMM: restricted maximum likelihood estimation and average marginal effects (AME)</u>

| **Terms** | **ML coefficients (SE)** | **p-values** | **BIC** | **Variance (intercept) Variance (slope)** | **Average marginal effects (AME)** |
|---|---|---|---|---|---|
| - Intercept | -0.46 (0.13) | 0 | -2745.3 | 0.09 | |
| - Time | -0.13 (0.009) | 0 | | 0.003 | -0.72% |
| - Restrictions on gatherings: gatherings of more than 100 people not permitted | -0.35 (0.08) | 0 | | | -2.58% |
| - Restrictions on gatherings: gatherings of between 11 and 100 people not permitted | -0.39 (0.08) | 0 | | | -2.78% |
| - Restrictions on gatherings: gatherings of 10 people or less not permitted | -0.39 (0.06) | 0 | | | -2.81% |
| | -0.24 (0.06) | 0 | | | -1.51% |
| - Workplace closing: require closing (or work from home) for some sectors or categories of workers | -0.29 (0.06) | 0 | | | -1.78% |
| - Workplace closing: require closing (or work from home) of all-but-essential workplaces (e.g. grocery stores, doctors) | | | | | |
| | -0.16 (0.07) | 0.02 | | | -1.12% |
| - School closing: require closing of only some levels or categories, e.g. just high school, or just public schools | -0.25 (0.08) | 0 | | | -1.65% |
| - School closing: require closing of all levels | | | | | |
| | -0.08 (0.05) | 0.09 | | | -0.45% |
| -Mask wearing requirements (mask wearing recommended) | -0.08 (0.04) | 0.04 | | | -0.44% |
| -Mask wearing requirements (mask wearing required in specific public spaces country-wide or in specific geographical areas) | -0.19 (0.06) | 0.003 | | | -0.96% |
| -Mask wearing requirements (mask wearing required country-wide in all public places or in all public places where social distancing is not possible) | -0.004 (0.001) | 0.001 | | | -0.02% |
| - Total number of tests performed per thousand population | | | | | |

The AME estimated based on the results of the mGLMM (last column), indicate that restrictions on gatherings had the highest impact of all NPIs in reducing the average daily growth rate in cumulative weekly confirmed COVID-19 cases (wADGR). Workplace closing requirements had the second highest impact, followed by school closing requirements. Mask wearing requirements ranked fourth in terms of impact and, as a proxy for testing strategy. Additionally, the total number of tests performed country-wide per thousand population was a significant predictor.

**Testing the assumptions of the mGLMM.**

In the sub-sections below we present our assessment of the model assumptions, including: adequacy of the probit link function, normality in the distribution of the random-effect residuals, no overdispersion (i.e. uniformity in the distribution of the scaled residuals and uniformity in y-direction of the residuals), no collinearity between the regressors.

*Adequacy of the probit link function*

Figure A4 presents, in the top panel, a plot of the observed and predicted wADGR (on the log-scale, for ease of visualization) for all countries and time points and, in the bottom panel, a posterior predictive check, i.e. a comparison of the observed response variable (red curve) with 250 simulations under the fitted model (grey area). From Figure 4, the mGLMM has a good predictive ability.

Figure A4. Observed versus predicted values of the wADGR for the mGLMM

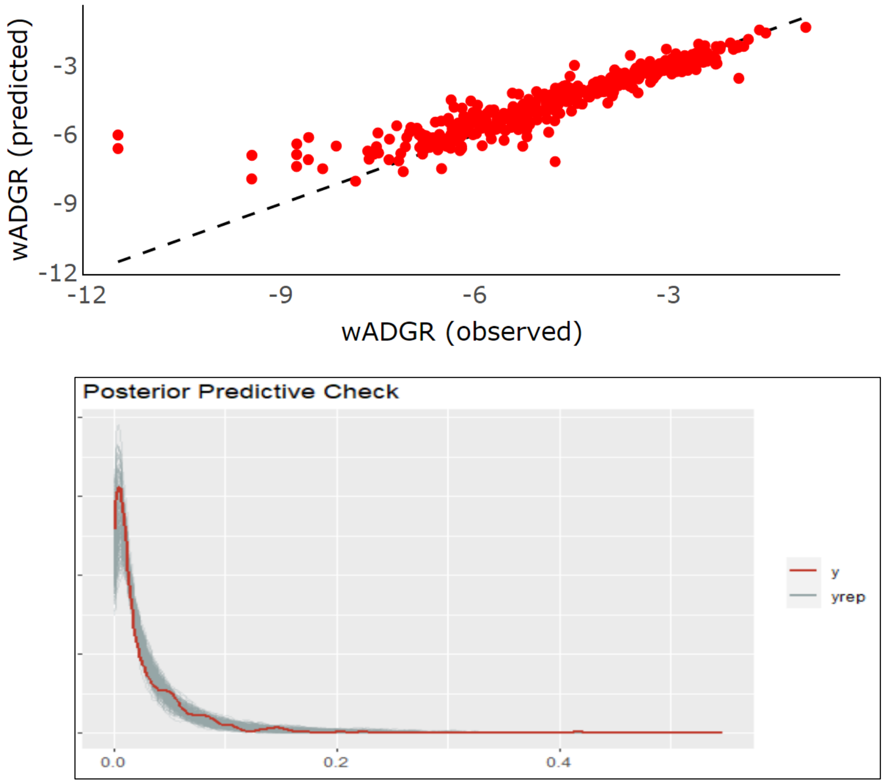

*Normality in the distribution of the random effects residuals*

Figure A5 shows two qqplots for, respectively, the random effects residuals of the mGLMM. From these plots, the distribution of these residuals is approximately Normal.

Figure A5. qqplots of the random effect residuals for the mGLMM

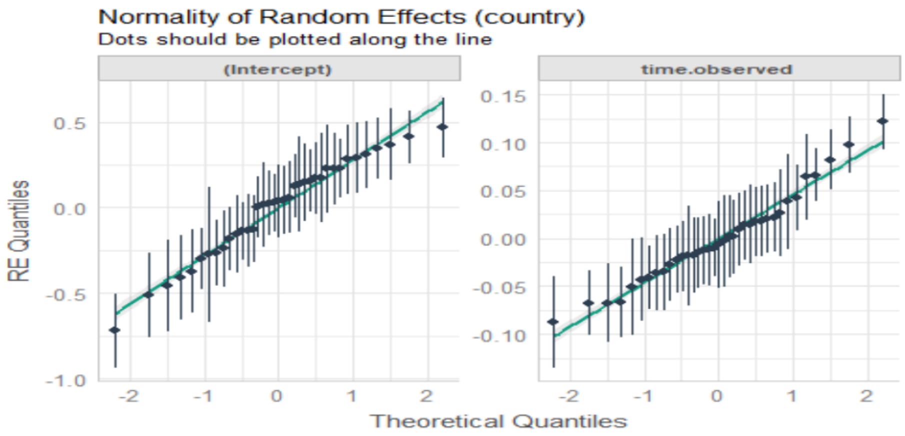


*No overdispersion*

Figure A6 shows two the output from the DHARMa package residual diagnostics. The left panel shows a qqplot comparing the distribution of the scaled residuals with their expected distribution. The scaled residuals of the mGLMM follow (as appropriate) a uniform distribution. The DHARMa package overdispersion test is not significant, the Kolmogorov-Smirnof test for correct distribution is not significant, the test for outliers is not significant. The right panel shows a plot of the empirical residuals in the 0.25, 0.5 and 0.75 quantiles against their predicted values to detect deviations from uniformity in y-direction. No significant deviations are detected.

Figure A6. Residual diagnostics for the mGLMM

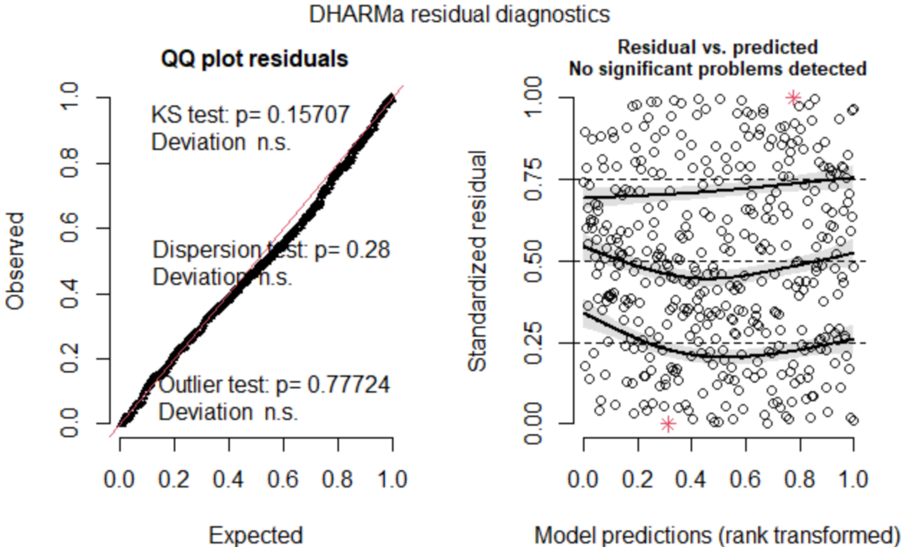

*Lack of strong collinearity between regressors*

Table A8 shows the variance inflation factor (VIF) for each of the regressors in the mGLMM. Values close to 1 indicate no collinearity.

Table A8. Variance inflation factor (VIF) for the mGLMM regressors

| **Regressors** | **VIF** |
|---|---|
| Time | 1.09 |
| Restrictions on gatherings | 1.16 |
| Mask wearing requirements | 1.24 |
| Workplace closing requirements | 1.14 |
| School closing requirements | 1.28 |
| Tests per thousand population | 1.05 |

### 3.1.3. Multivariable generalised linear mixed model (mGLMM) with NPI-time interactions

For the mGLMM, we explored the interactions between the NPIs and the time variable to understand what policies may impact longitudinal changes in the wADGR. Table A9 shows the results of the mGLMM with NPI-time interactions adjusted for overdispersion in workplace closing requirements. The best fitting model showed an interaction of mask wearing requirements with time.

<u>Table A9. mGLMM with mask wearing requirements-time interaction: restricted maximum likelihood estimation and average marginal effects (AME)</u>

| **Terms** | **ML coefficients (SE)** | **p-values** | **BIC** | **Variance (intercept) Variance (slope)** | **Average marginal effects (AME)** |
|---|---|---|---|---|---|
| - Intercept | -0.54 (0.13) | 0 | -2747.1 | 0.09 | |
| - Time | -0.17 (0.011) | 0 | | 0.003 | -0.78% |
| - Restrictions on gatherings: gatherings of more than 100 people not permitted | -0.30 (0.08) | 0 | | | -2.04% |
| - Restrictions on gatherings: gatherings of between 11 and 100 people not | -0.30 (0.08) | 0 | | | -2.07% |
| permitted | -0.33 (0.06) | 0 | | | -2.24% |
| - Restrictions on gatherings: gatherings of 10 people or less not permitted | | | | | |
| | -0.15 (0.06) | 0.006 | | | -0.92% |
| - Workplace closing: require closing (or work from home) for some sectors or categories of workers | -0.19 (0.06) | 0 | | | -1.11% |
| - Workplace closing: require closing (or work from home) of all-but-essential workplaces (e.g. grocery stores, doctors) | | | | | |
| | -0.19 (0.07) | 0.008 | | | -1.26% |
| - School closing: require closing of only some levels or categories, e.g. just high school, or just public schools | -0.24 (0.08) | 0.02 | | | -1.57% |
| - School closing: require closing of all levels | | | | | |
| | -0.10 (0.08) | 0.21 | | | -0.25% |
| -Mask wearing requirements (mask wearing recommended) | -0.29 (0.05) | 0 | | | -0.35% |
| -Mask wearing requirements (mask wearing required in specific public spaces country-wide or in specific geographical areas) | -0.22 (0.11) | 0.04 | | | -0.65% |
| -Mask wearing requirements (mask wearing required country-wide in all public places or in all public places where social distancing is not possible) | 0.02 (0.02) | 0.22 | | | |
| | 0.08 (0.01) | 0 | | | |
| - Interaction Mask-wearing 1 – time | 0.04 (0.02) | 0.10 | | | |
| - Interaction Mask-wearing 2 – time | | | | | |
| - Interaction Mask-wearing 3 - time | -0.005 (0.001) | 0 | | | -0.02% |
| - Total number of tests performed per thousand population | | | | | |

The AME estimated based on the results of the mGLMM with a mask wearing requirements-time interaction (last column) indicate, again, as was the case with the mGLMM without interactions, that restrictions on gatherings had the highest impact of all NPIs in reducing the wADGR. Comparing the last column of Table A9 with the last column of Table A7, the interaction of mask wearing requirements with time results in a slight decrement of the average marginal effects of the NPIs.

**Testing the assumptions of the mGLMM with mask wearing requirements-time interaction.**

As with the mGLMM with no interactions, in the sub-sections below we present our assessment of the model assumptions (except collinearity, as the model incorporates interaction terms).

*Adequacy of the probit link function*

Figure A7 presents, in the top panel, a plot of the observed and predicted log-scaled wADGR for all countries and time points and, in the bottom panel, a posterior predictive check. From Figure 4, the mGLMM with mask wearing requirements-time interaction has a good predictive ability.

Figure A7. Observed versus predicted values of the wADGR for the mGLMM with mask wearing requirements-time interaction

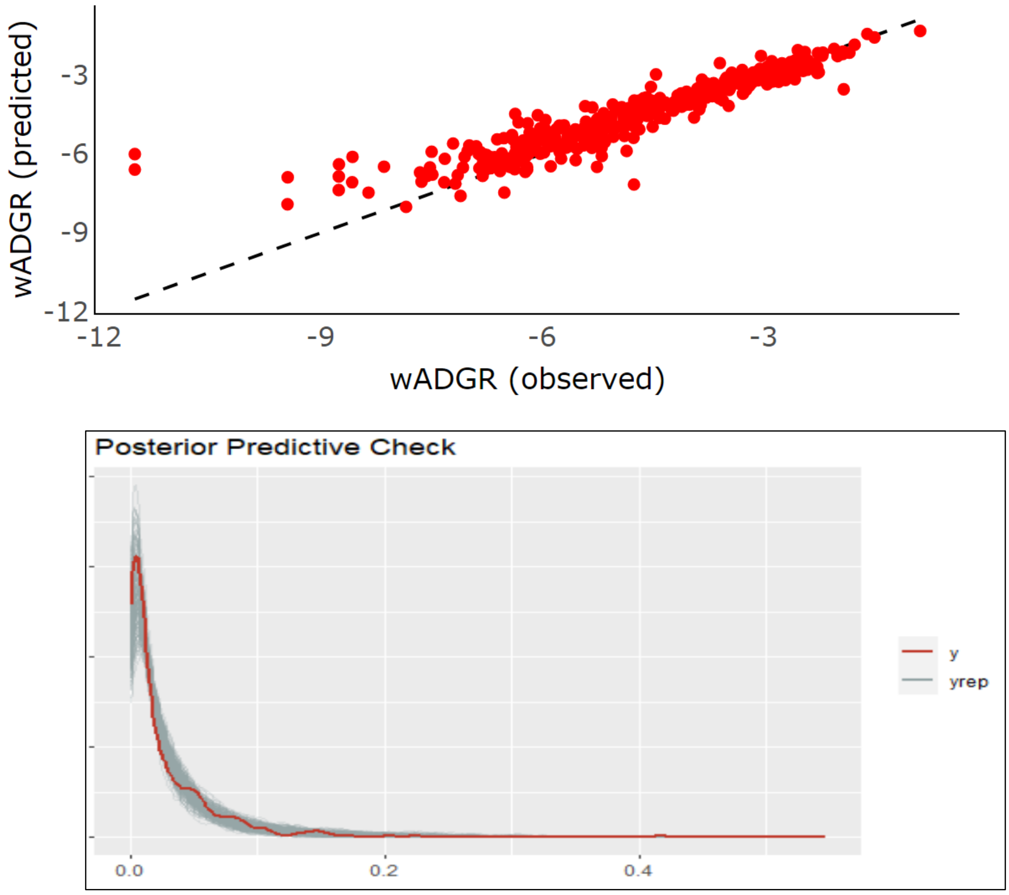

*Normality in the distribution of the random effects residuals*

Figure A8 shows two qqplots for, respectively, the random effects residuals of the mGLMM with interaction between mask wearing requirements and time. From these plots, the distribution of these residuals is approximately Normal.

Figure A8. qqplots of the random effect residuals for the mGLMM with mask wearing requirements-time interaction.

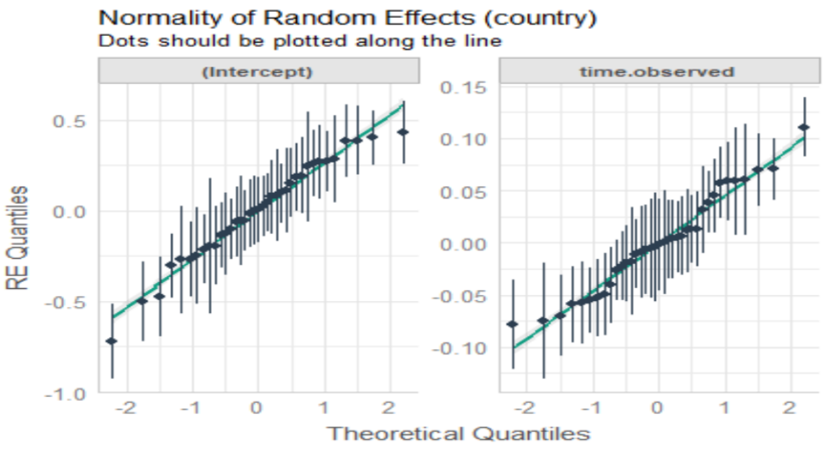


*No overdispersion*

Figure A9 shows the output from the DHARMa package residual diagnostics. The scaled residuals of the mGLMM follow (as appropriate) a uniform distribution. The DHARMa package overdispersion test is not significant, the Kolmogorov-Smirnof test for correct distribution is not significant, the test for outliers is not significant. No significant deviations from uniformity in y-direction are detected.

Figure A9. Residual diagnostics for the mGLMM with mask wearing requirement-time interaction

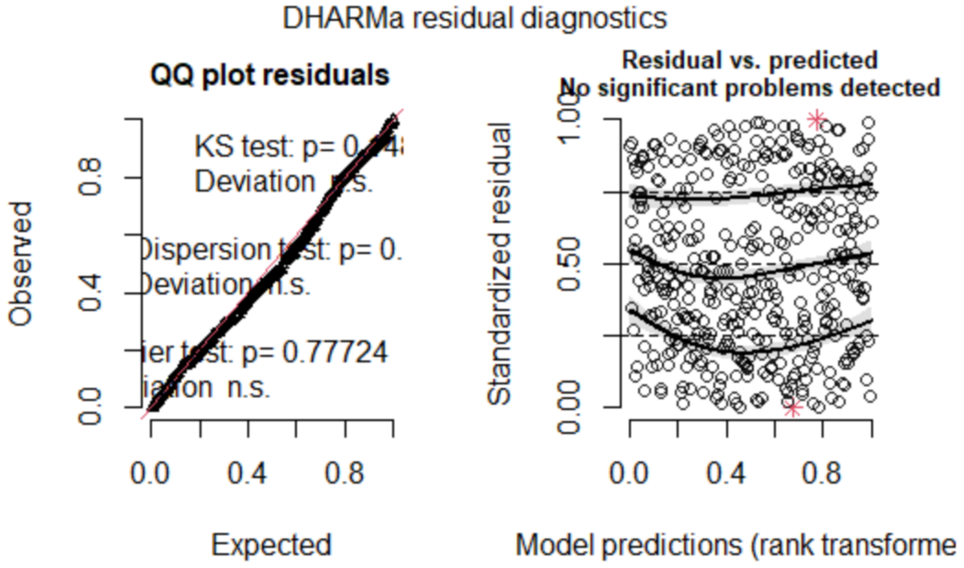

### 3.2. Analysis of the period October 1-December 31, 2020.

Figure A10 below presents side by side the change over time in the intensity of the NPIs across OECD countries for the period October-December 2020 and for the initial phase of the epidemic (in each graph, each point is the intensity of the corresponding intervention in each country during the relevant week).

<u>Figure A10. Intensity of individual NPIs over time in OECD member states. Top panel: 11 weeks in the period October-December 2020. Bottom panel: 11 weeks in the early stage of the epidemic</u>

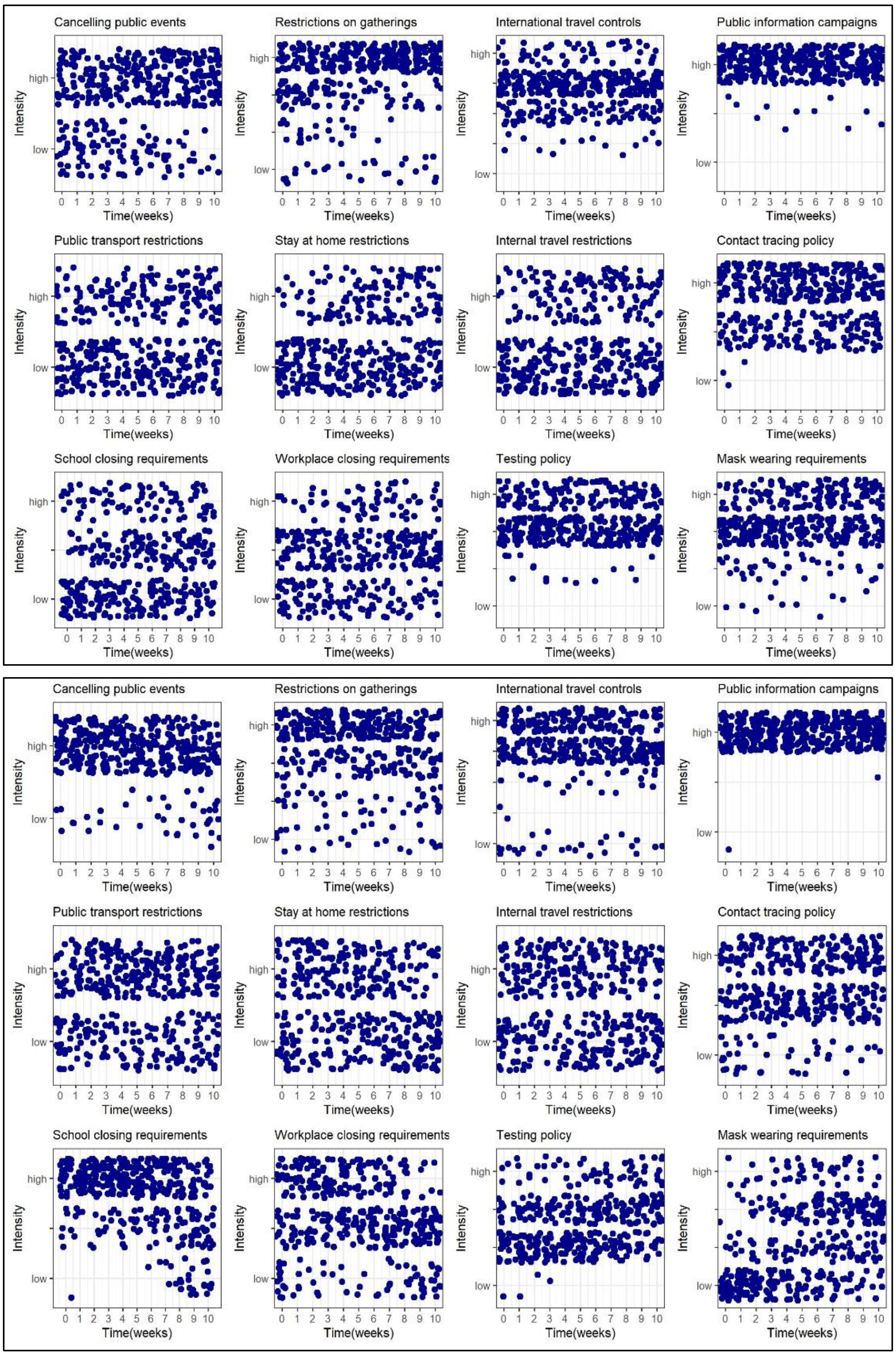

From Figure A10, there were several differences in the intensity of each NPI in the period October-December 2020 compared to the initial stages of the epidemic:

1. Public event cancelling requirements. In the period October-December 2020 (P2), this policy was overall less stringent but tightened more over time than in the study period of the initial epidemic stage (P1)

2. Restrictions on gatherings. In P2, this NPI was implemented at a higher level of stringency and tightened over time compared to P1

3. International travel controls. In P2 this NPI was stable over time and overall less stringent than in P1.

4. Public information campaigns. In P2, this NPI was stable over time and overall slightly more intense than in P1

5. Public transport restrictions. In P2, this NPI intensified slightly over time and was overall less stringent than in P1

6. Stay at home restrictions. In P2, this NPI tightened somewhat over time and was overall less stringent than in P1

7. Internal travel restrictions. In P2, this NPI was stable over time and overall somewhat less stringent than in P1

8. Contact tracing policy. In P2, this NPI was stable over time and overall more intense than in P1.

9. School closing requirements. In P2, this NPI tightened slightly over time and was overall less stringent than in P1

10. Work closing requirements. In P2, this NPI tightened over time and was slightly less stringent than in P1

11. Testing policy. In P2, this NPI was stable over time and was overall more intense than in P1

12. Mask wearing requirements. In P2, this NPI tightened slightly over time and was overall more stringent than in P1

### 3.2.1 Multivariable linear mixed model (mLMM2)

**Outcome and overall approach to model fitting.**

We performed a probit transformation of the ADGR values (probit_wADGR). We then fitted a series of OLS-lines to the probit_ADGR trajectories (see Figure A11). From Figure A11, it was appropriate to use a growth model linear in time.

Figure A11. Probit ADGR trajectories and fitted OLS lines.

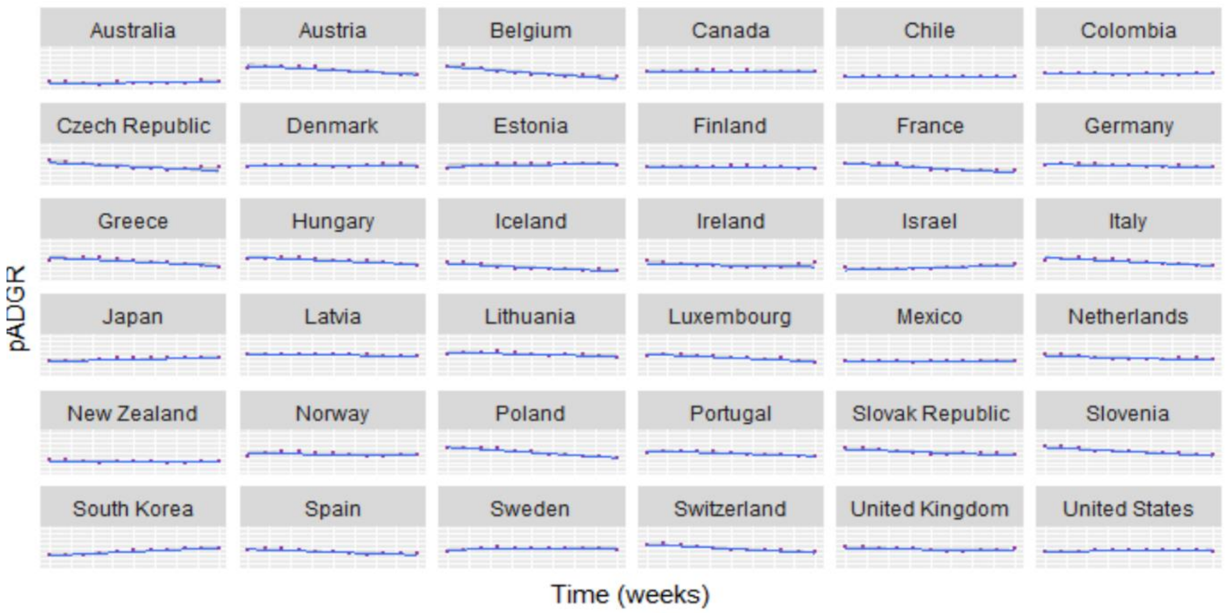


**Identifying the mLMM2: forward selection.**

Table A10 below shows the results of the univariate linear mixed models (only for the NPIs which showed a statistical effect) and their rank. This rank determined the sequence in which the regressors were introduced into the forward selection models:

Table A10. Univariate results and sequence in which the regressors were introduced into the forward selection models.

| Model (rank) | Regressors | Coefficients (SE) | p-values | BIC | Variance (intercept)<br>Variance (slope)<br>Variance (residuals) |
|---|---|---|---|---|---|
| Model 1<br>(1) | -Intercept<br>-Time<br>-Workplace closing (require closing or work from home for some sectors or categories of workers)<br>-Workplace closing (require closing or work from home all-but-essential workplaces e.g. grocery stores, doctors) | -2.03 (0.08)<br>-0.03 (0.008)<br>-0.04 (0.03)<br><br><br>-0.21 (0.04) | 0<br>0<br>0.16<br><br><br>0 | -266.2 | 0.19<br>0.002<br>0.014 |
| Model 2<br>(2) | -Intercept<br>-Time<br>-Restrictions on internal movement (internal movement restrictions in place) | -2.04 (0.07)<br>-0.03 (0.008)<br>-0.10 (0.03) | 0<br>0<br>0 | -239.4 | 0.18<br>0.002<br>0.015 |
| Model 3<br>(3) | -Intercept<br>-Time<br>-Stay at home requirements (require not leaving house with<br>exceptions for daily exercise, grocery shopping, and 'essential' trips or require not leaving house with minimal exceptions e.g. allowed to leave only once a week, or only one person can leave at a time, etc.) | -2.06 (0.07)<br>-0.03 (0.008)<br>-0.074 (0.026) | 0<br>0<br>0.005 | -234.6 | 0.14<br>0.002<br>0.016 |
| Model 4<br>(4) | -Intercept<br>-Time<br>- School closing (only some levels or categories, e.g. just high school, or just public schools)<br>- School closing (require closing all levels) | -2.05 (0.07)<br>-0.03 (0.007)<br>-0.05 (0.03)<br><br>-0.09 (0.03) | 0<br>0<br>0.06<br><br>0.009 | -228.5 | 0.19<br>0.002<br>0.016 |
| Model 5<br>(5) | -Intercept<br>-Time<br><br>-Testing of anyone showing COVID-19 symptoms<br><br>-open public testing (e.g. "drive through" testing available to asymptomatic people) | -2.25 (0.11)<br>-0.03 (0.008)<br><br>0.19 (0.08)<br><br><br>0.18 (0.09) | 0<br>0<br><br>0.02<br><br><br>0.045 | -226 | 0.19<br>0.002<br>0.016 |

Table A11 below shows the mLMM2 resulting from the forward selection process.

Table A11. mLMM2: maximum likelihood and Bayesian estimation.

| Model | Regressors | ML coefficients (SE) | p-values | BIC | Variance (intercept) Variance (slope) Variance (residuals) | Mean coefficients from Bayesian estimation |
|---|---|---|---|---|---|---|
| Model 6 | -Intercept<br>-Time | -1.22 (0.32)<br>-0.03 (0.008) | 0<br>0 | -261.4 | 0.14<br>0.002<br>0.014 | -0.65<br>-0.03 |
| | -Workplace closing (require closing or work from home for some sectors or categories of workers) | -0.04 (0.03) | 0.15 | | | -0.03 |
| | -Workplace closing (require closing or work from home all-but-essential workplaces e.g. grocery stores, doctors) | -0.21 (0.04) | 0 | | | -0.20 |
| | -Testing of anyone showing COVID-19 symptoms | 0.17 (0.08) | 0.03 | | | 0.19 |
| | -open public testing (e.g. “drive through” testing available to asymptomatic people) | 0.14 (0.08) | 0.10 | | | 0.13 |
| | - Percentage of total population living in urban areas | -0.01 (0.004) | 0.003 | | | -0.02 |

**Testing the assumptions of the mLMM2.**

In the sub-sections below we present our assessment of the model assumptions, including: Normality in the residuals, heteroscedasticity in the residuals, and lack of strong collinearity in the regressors.

*Normality in the distribution of the residuals*

Figure A12 presents qqplots for, respectively, the level-1 residuals, i.e. the residuals across all countries and all occasions of measurement (top graph) and the level-2 residuals, i.e. the residuals in the intercepts and slopes across countries (bottom graph), i.e. in the between-country intercept and slope residuals. From Figure A12, the distribution of these residuals can be considered approximately Normal.

Figure A12. Level-1 residuals and Level-2 residuals for the mLMM2: qqplots

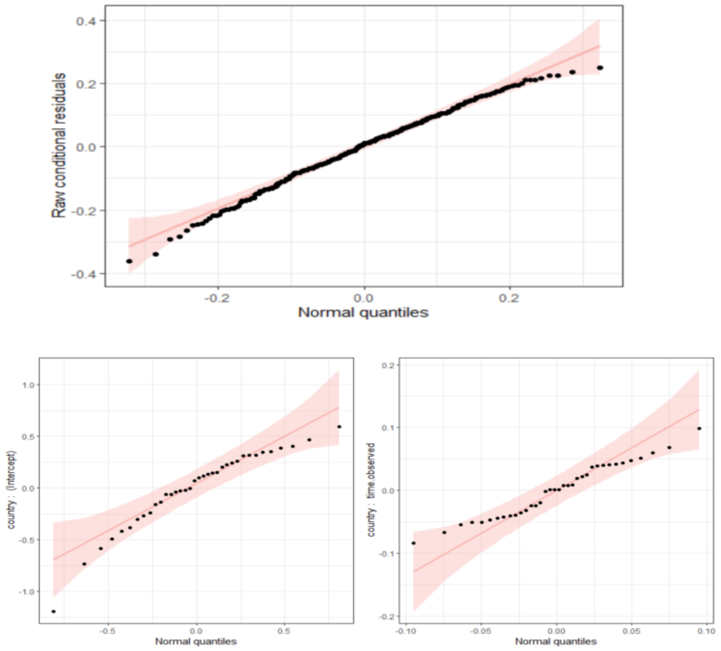


*Homoscedasticity in the distribution of the residuals*

Figure A13 (top graph) maps the studentized level-1 residuals against the final model's fitted values, showing an approximately constant variance of these residuals around zero across the predicted values for the outcome. Figure A13 (bottom graph) plots the distribution of these residuals at different time points, showing approximately constant variance across time.

Figure A13. Level-1 residuals for the mLMM2: homoscedasticity

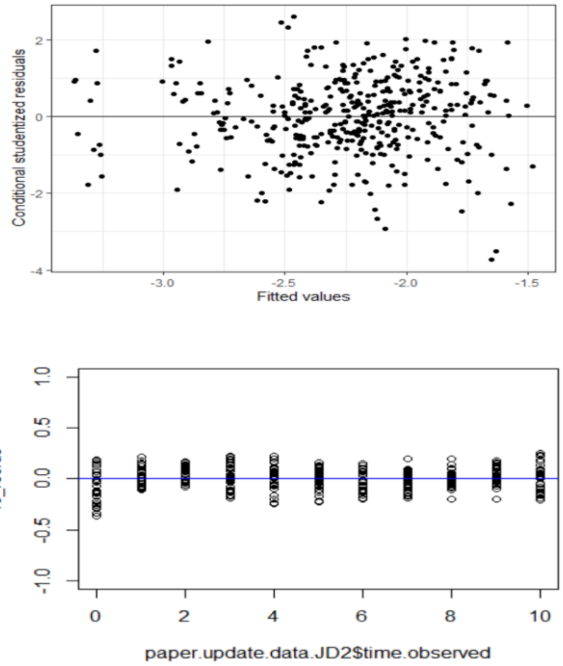

*Lack of strong collinearity between regressors*

Table A12 shows the variance inflation factor (VIF) for each of the regressors in the mLMM2. Values close to 1 indicate no collinearity.

Table A12. Variance inflation factor (VIF) for the mLMM2 regressors

| Regressors | GVIF^(1/(2*Df)) |
|---|---|
| Time | 1.01 |
| Work closing | 1 |
| Testing policy | 1 |
| Percentage of total population living in urban areas | 1 |

### 3.1.2. Multivariable generalised linear mixed model (mGLMM2)

We fitted a multivariable beta regression generalized linear mixed model (mGLMM2) with a probit link function using the average daily rate of growth in the cumulative weekly cases (wADGR) as the response variable. We used restricted maximum likelihood estimation to fit the model. The mGLMM2 allowed for the estimation of the average marginal effects (AME) of the NPIs on the wADGR. The AME provide a measure, across all observed data, of the average change in the wADGR which results from changes in the level of intensity of each of the NPIs. The best fitting mGLMM2 was adjusted by modelling the model dispersion with test policy requirements as a regressor. Table A13 presents the model results.

Table A13. mGLMM2: restricted maximum likelihood estimation and average marginal effects (AME)

| **Terms** | **ML coefficients (SE)** | **p-values** | **BIC** | **Variance (intercept) Variance (slope)** | **Average marginal effects (AME)** |
|---|---|---|---|---|---|
| -Intercept<br>-Time | -1.38 (0.29)<br>-0.03 (0.01) | 0<br>0 | -2914 | 0.11<br>0.001 | <br>-0.13% |
| -Workplace closing (require closing or work from home for some sectors or categories of workers) | -0.01 (0.026) | 0.77 | | | -0.03% |
| -Workplace closing (require closing or work from home all-but-essential workplaces e.g. grocery stores, doctors) | -0.18 (0.037) | 0 | | | -0.66% |
| -Testing of anyone showing COVID-19 symptoms | 0.28 (0.06) | 0 | | | 0.89% |
| -open public testing (e.g. “drive through” testing available to asymptomatic people) | 0.26 (0.07) | 0 | | | 0.83% |
| - Percentage of total population living in urban areas | -0.01 (0.04) | 0.001 | | | -0.05% |

**Testing the assumptions of the mGLMM2.**

We tested the following model assumptions: adequacy of the probit link function, normality in the distribution of the random-effect residuals, no overdispersion (i.e. uniformity in the distribution of the scaled residuals and uniformity in y-direction of the residuals), no collinearity between the regressors.

*Adequacy of the probit link function*

Figure A14 presents, in the top panel, a plot of the observed and predicted wADGR (on the log-scale, for ease of visualization) for all countries and time points and, in the bottom panel, a posterior predictive check, i.e. a comparison of the observed response variable (red curve) with 250 simulations under the fitted model (grey area). From Figure A14, the mGLMM2 has a good predictive ability.

Figure A14. Observed versus predicted values of the wADGR for the mGLMM2

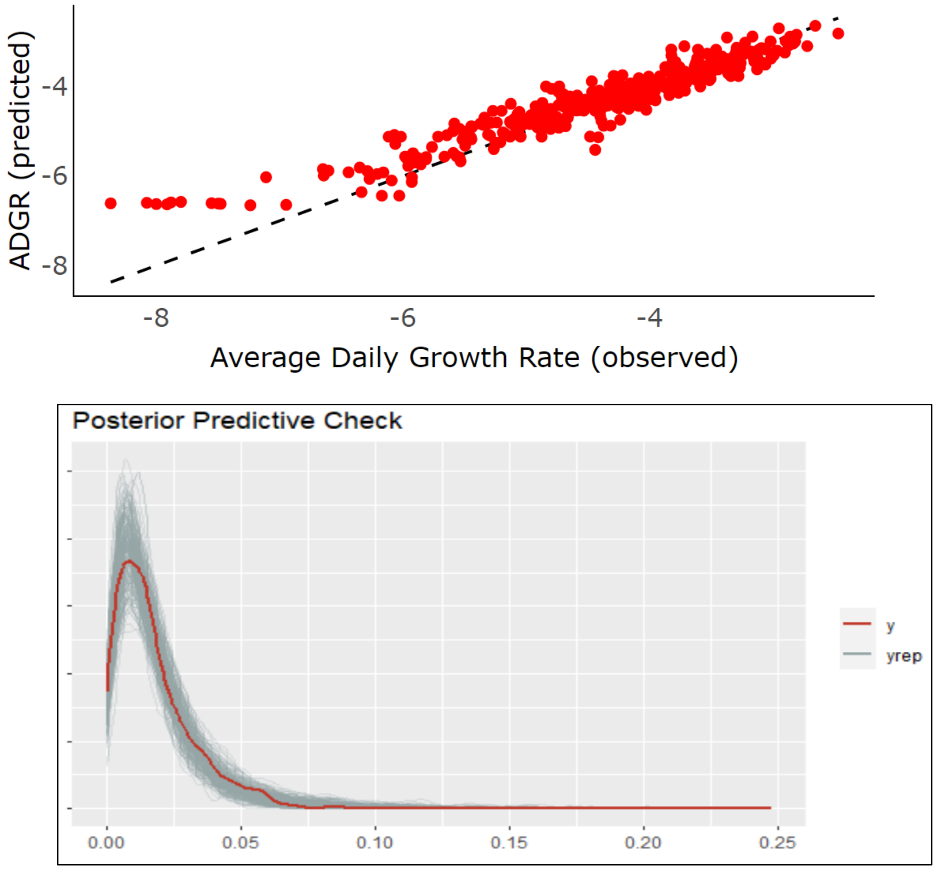


*Normality in the distribution of the random effects residuals*

Figure A15 shows two qqplots for, respectively, the random effects residuals of the mGLMM2. From these plots, the distribution of these residuals is approximately Normal.

Figure A15. qqplots of the random effect residuals for the mGLMM2

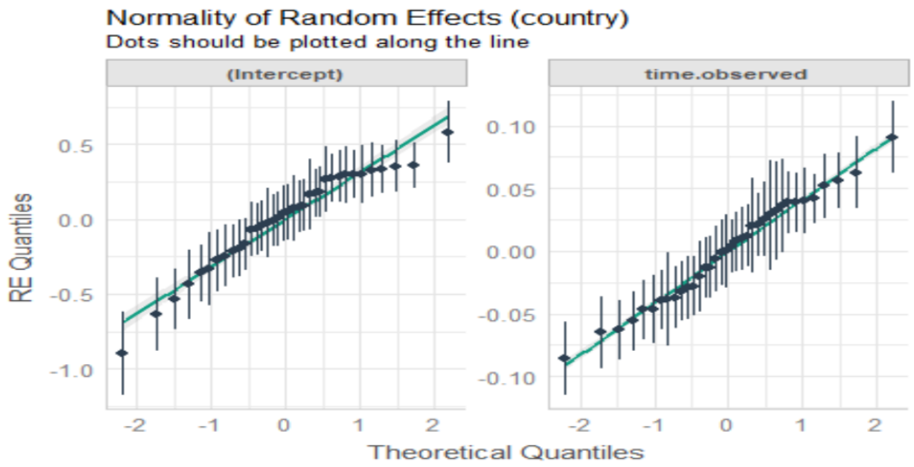

*No overdispersion*

Figure A16 shows two the output from the DHARMa package residual diagnostics. The left panel shows a qqplot comparing the distribution of the scaled residuals with their expected distribution. The scaled residuals of the mGLMM follow (as appropriate) a uniform distribution. The DHARMa package overdispersion test is not significant, the Kolmogorov-Smirnof test for correct distribution is not significant, the test for outliers is not significant. The right panel shows a plot of the empirical residuals in the 0.25, 0.5 and 0.75 quantiles against their predicted values to detect deviations from uniformity in y-direction. Although some significant deviations are detected at two quantiles, the combined adjusted quantile test is not significant.

Figure A16. Residual diagnostics for the mGLMM2

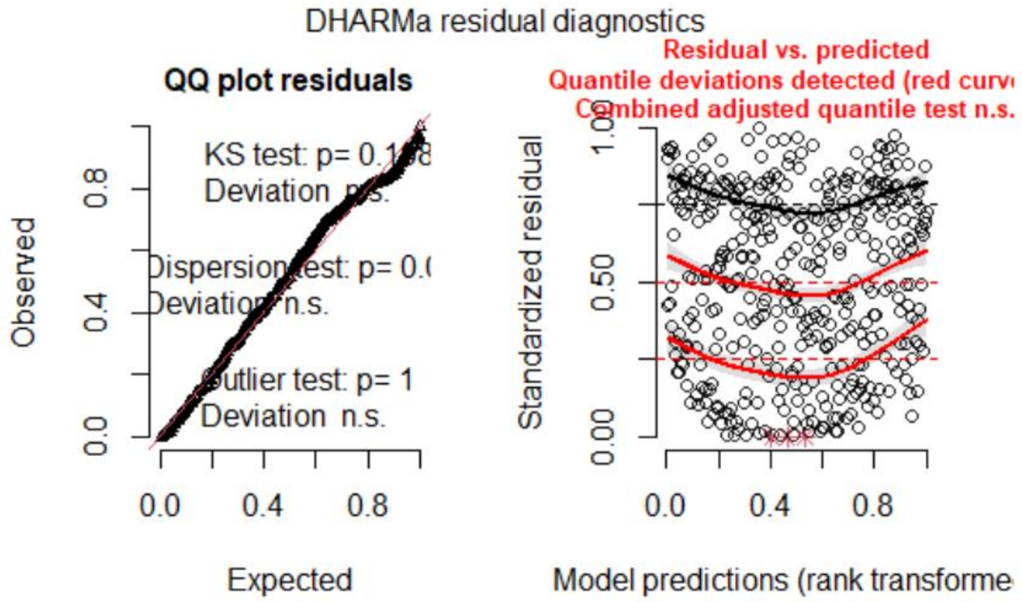

**Appendix 4. Distributions of the levels of intensity of NPIs across time across all countries: Initial epidemic phase**

1. Distribution of the levels of intensity over time across all countries: public event cancelling

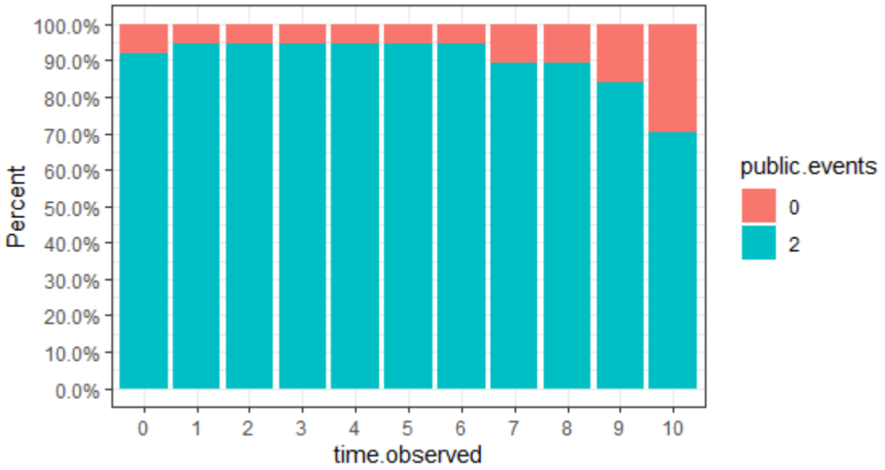


2. Distribution of the levels of intensity over time across all countries: restrictions on gatherings

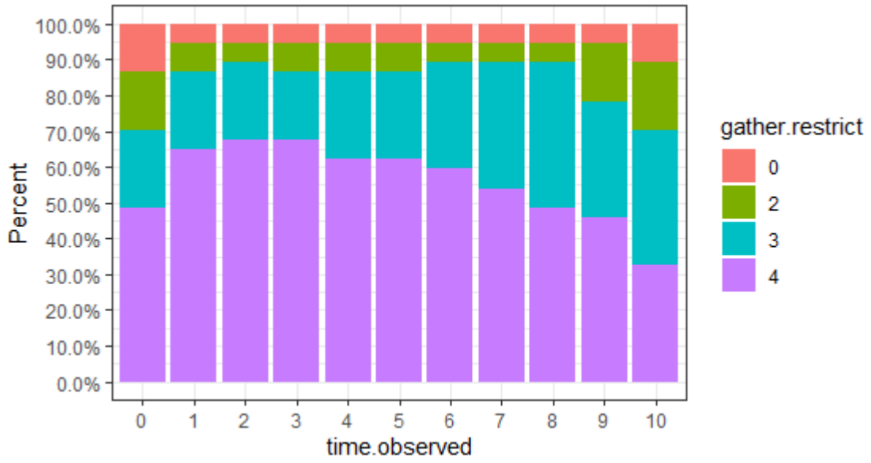


3. Distribution of the levels of intensity over time across all countries: international travel controls

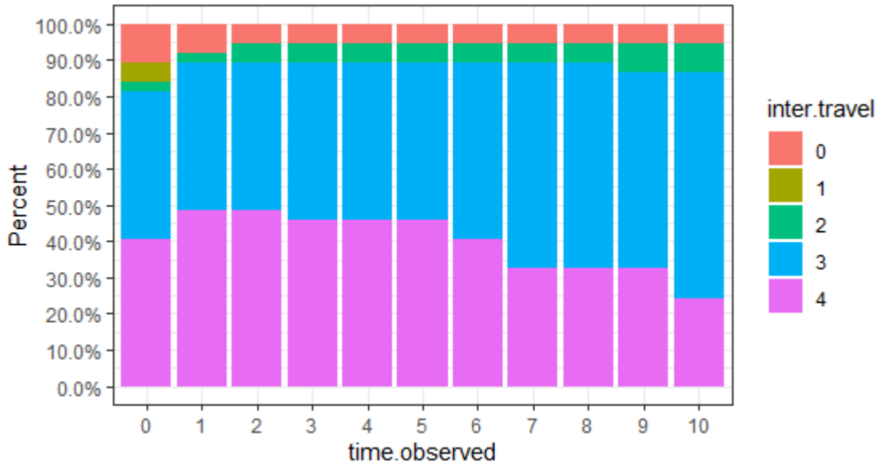

4. Distribution of the levels of intensity over time across all countries: public information campaigns

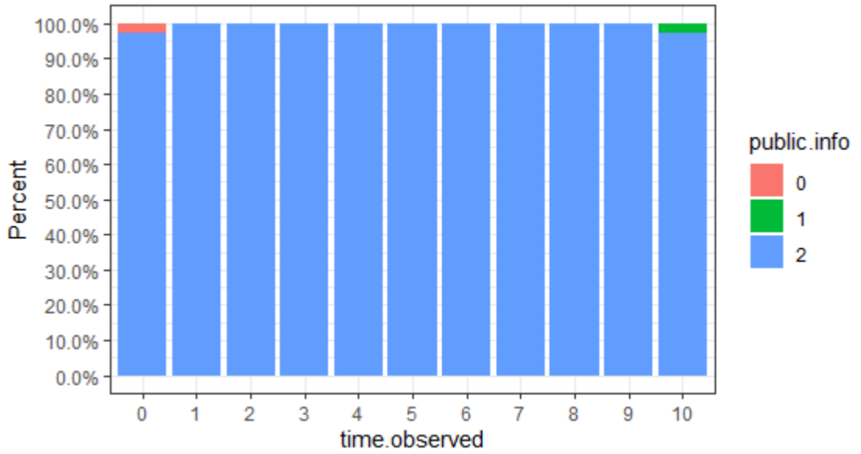


5. Distribution of the levels of intensity over time across all countries: public transport restrictions

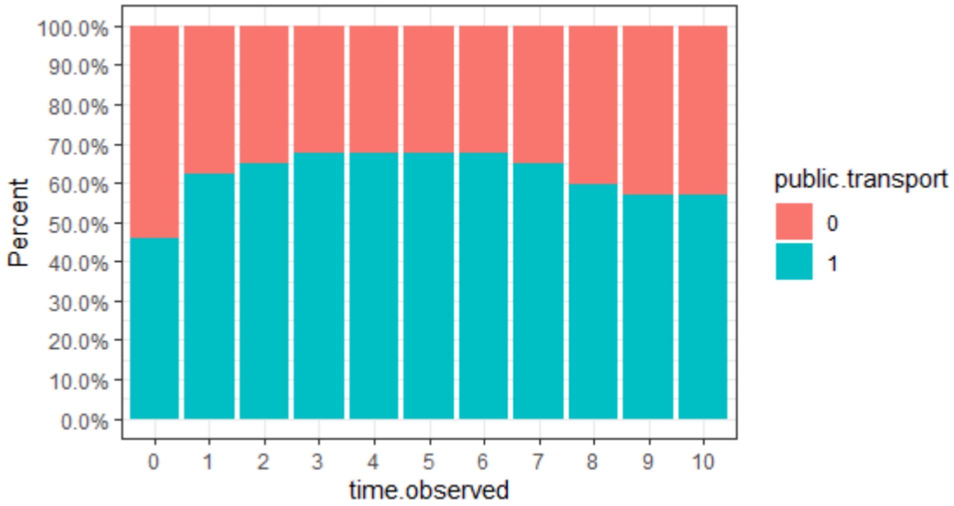


6. Distribution of the levels of intensity over time across all countries: stay at home restrictions

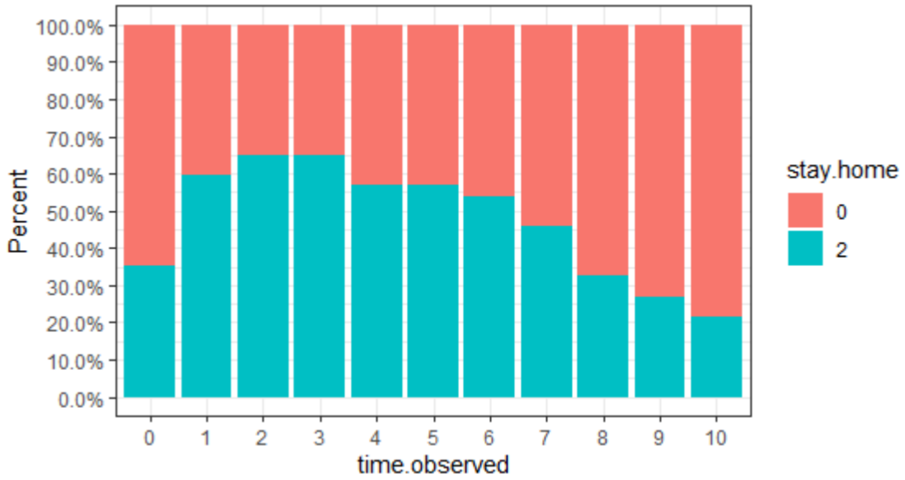

7. Distribution of the levels of intensity over time across all countries: internal travel restrictions

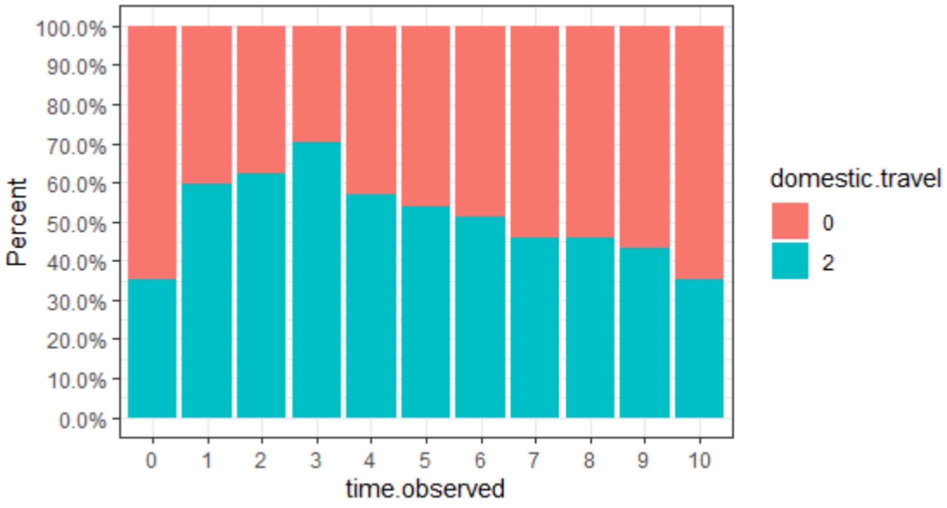


8. Distribution of the levels of intensity over time across all countries: contact tracing policy

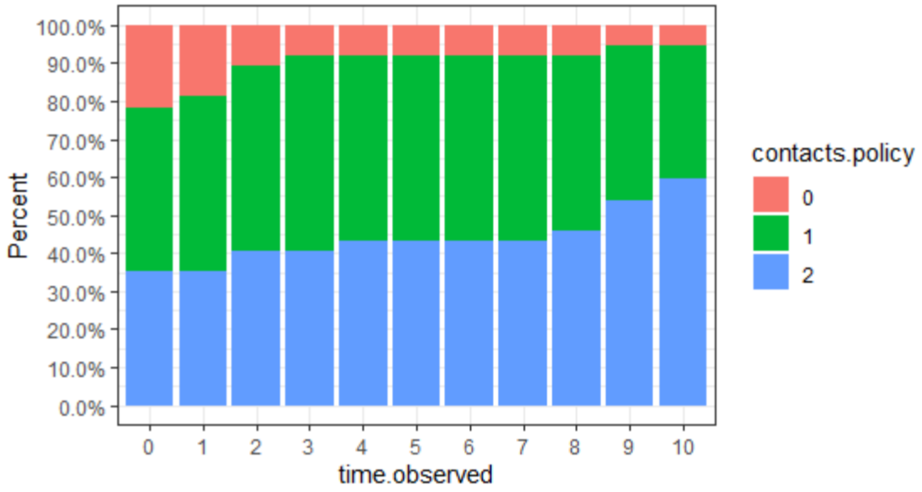


9. Distribution of the levels of intensity over time across all countries: school closing requirements

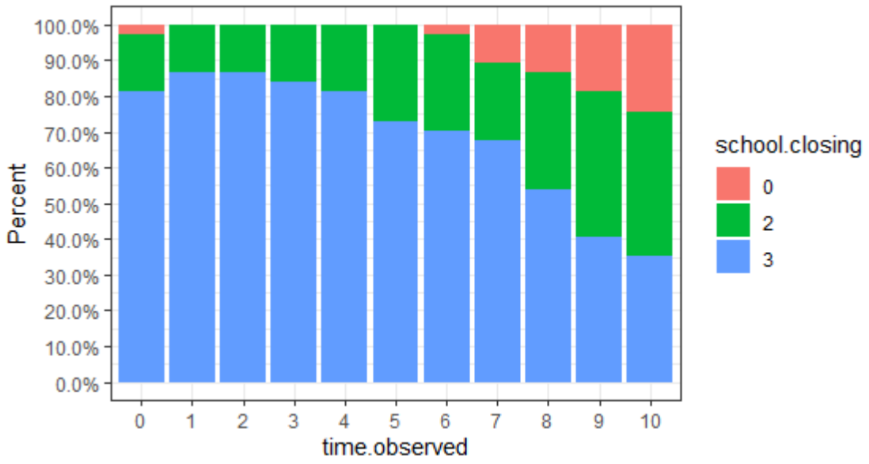

10. Distribution of the levels of intensity over time across all countries: workplace closing requirements

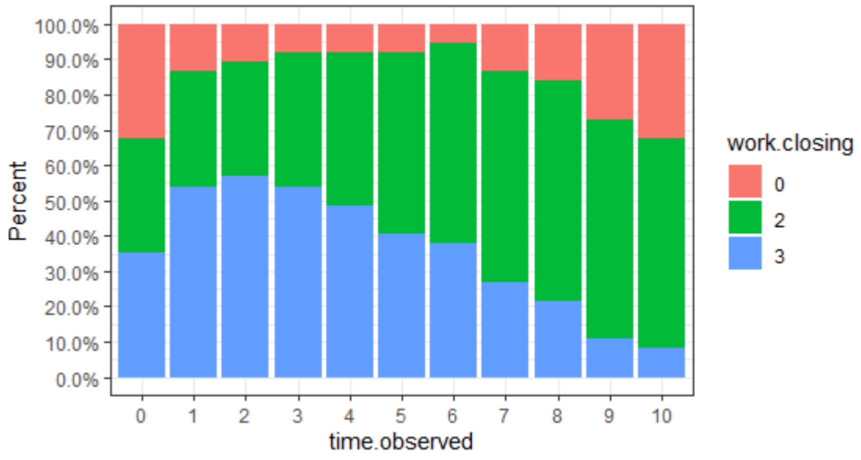


11. Distribution of the levels of intensity over time across all countries: testing policy

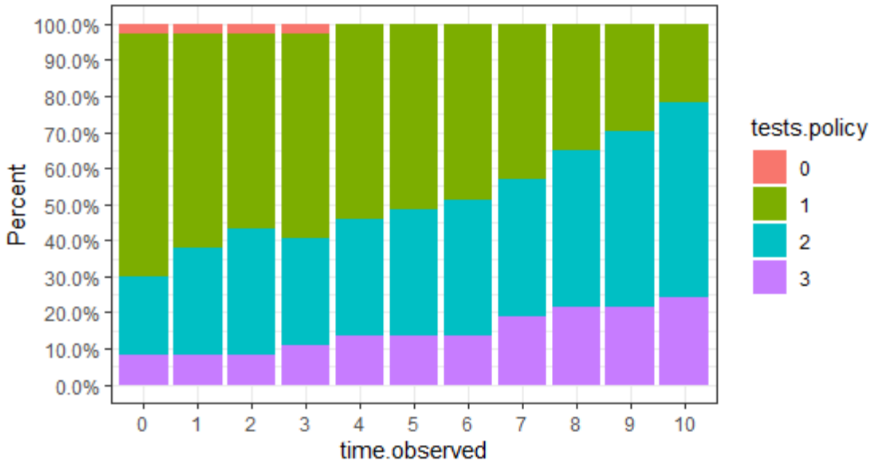


12. Distribution of the levels of intensity over time across all countries: mask wearing requirements

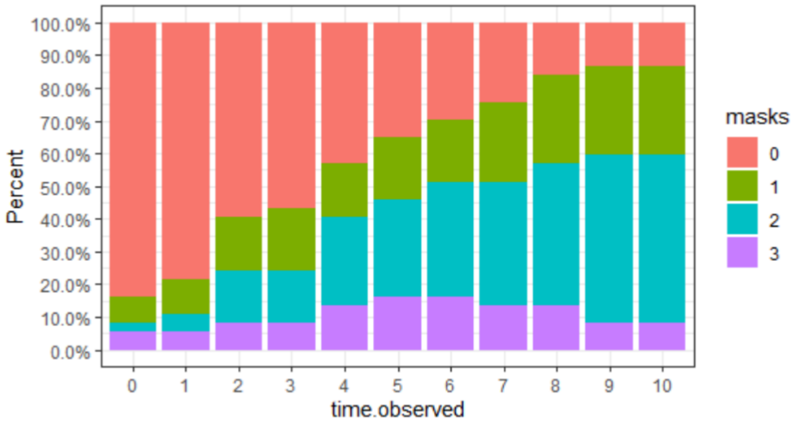

**Appendix 5. Distributions of the levels of intensity of NPIs across time across all countries: October-December 2020**

1. Distribution of the levels of intensity over time across all countries: public event cancelling

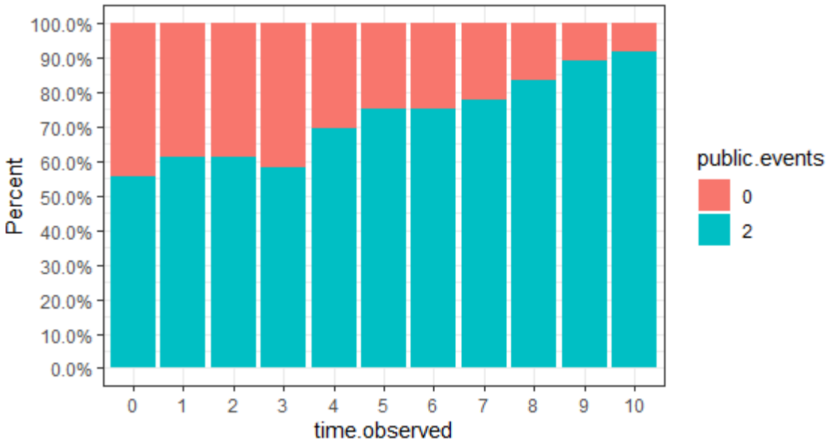


2. Distribution of the levels of intensity over time across all countries: restrictions on gatherings

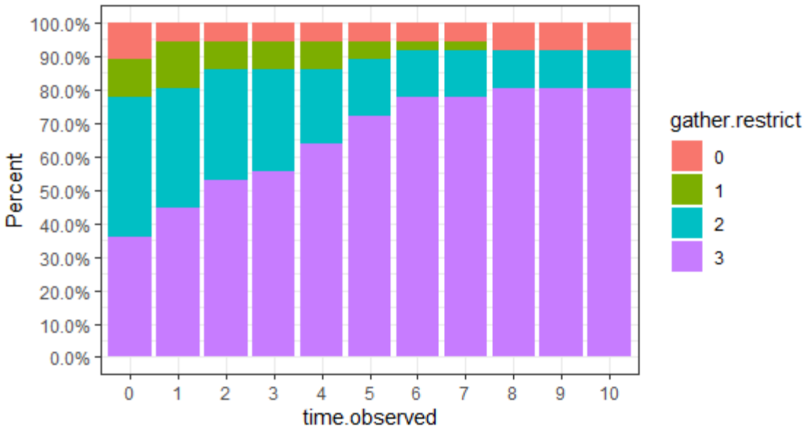


3. Distribution of the levels of intensity over time across all countries: international travel controls

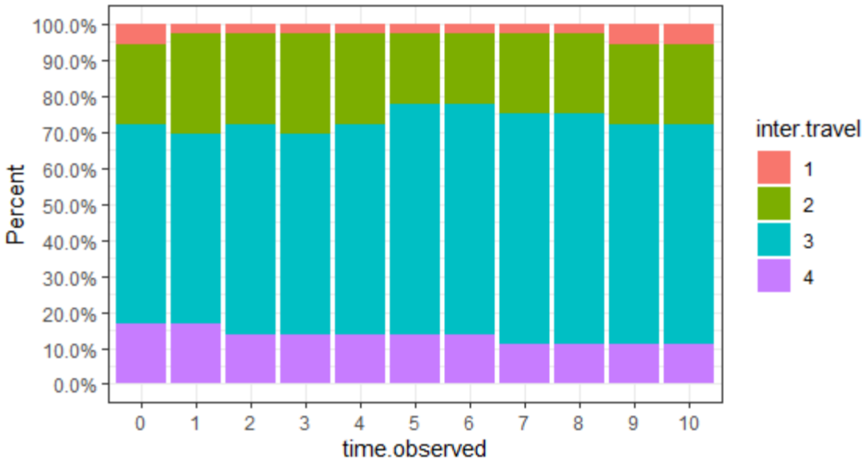

4. Distribution of the levels of intensity over time across all countries: public information campaigns

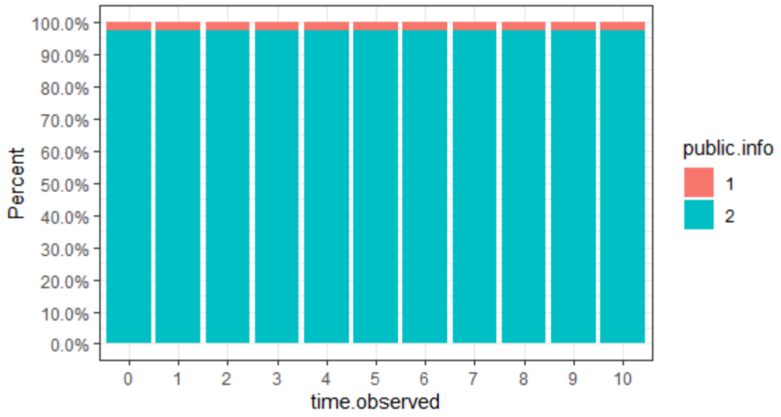


5. Distribution of the levels of intensity over time across all countries: public transport restrictions

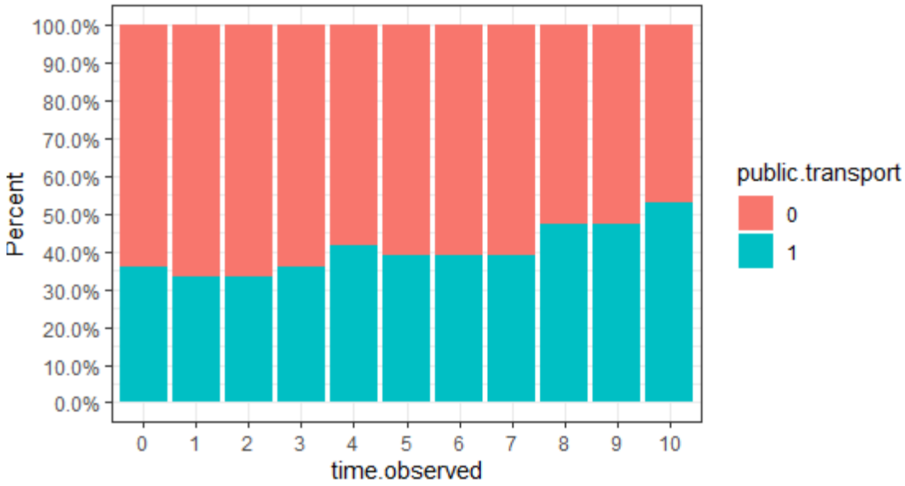


6. Distribution of the levels of intensity over time across all countries: stay at home restrictions

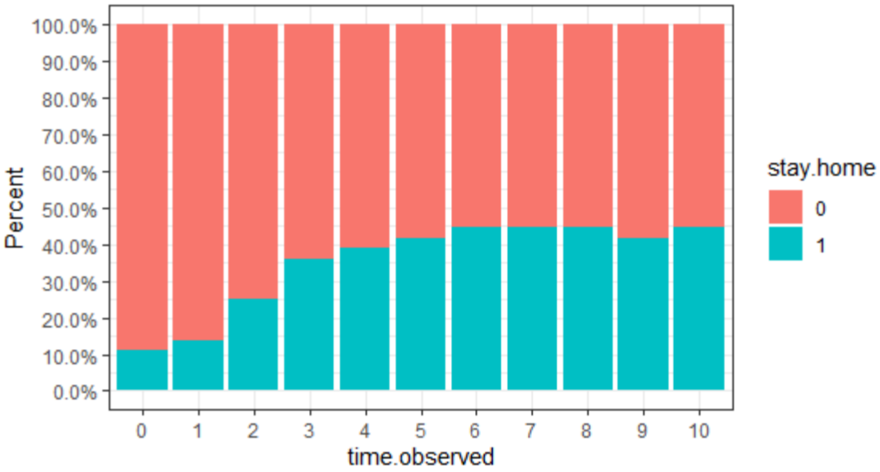

7. Distribution of the levels of intensity over time across all countries: internal travel restrictions

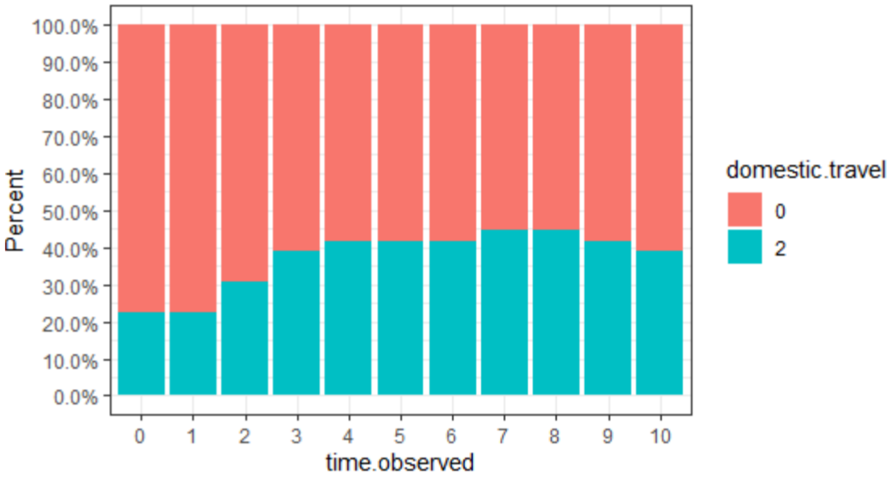


8. Distribution of the levels of intensity over time across all countries: contact tracing policy

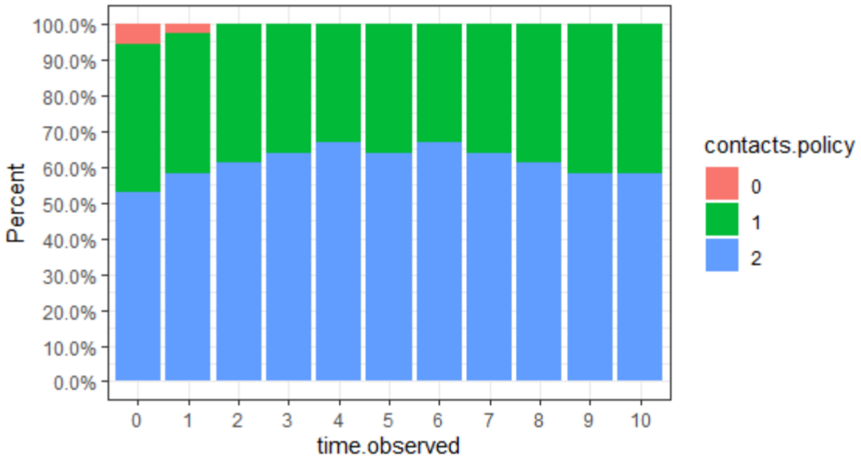


9. Distribution of the levels of intensity over time across all countries: school closing requirements

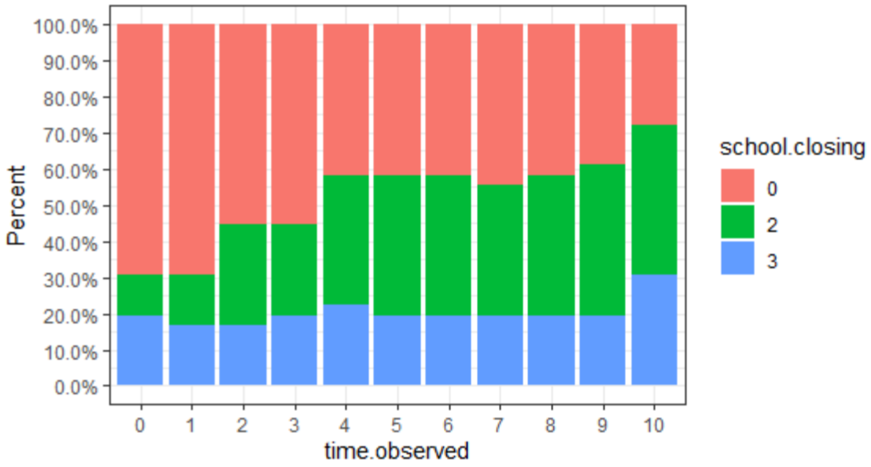

10. Distribution of the levels of intensity over time across all countries: workplace closing requirements

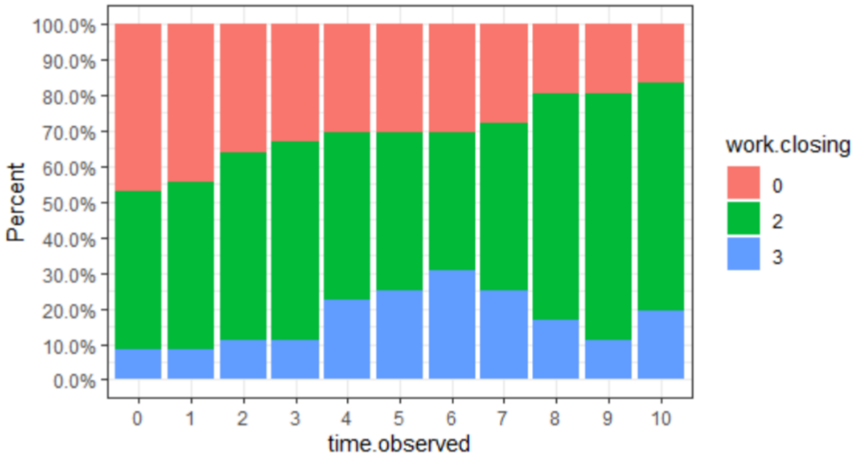


11. Distribution of the levels of intensity over time across all countries: testing policy

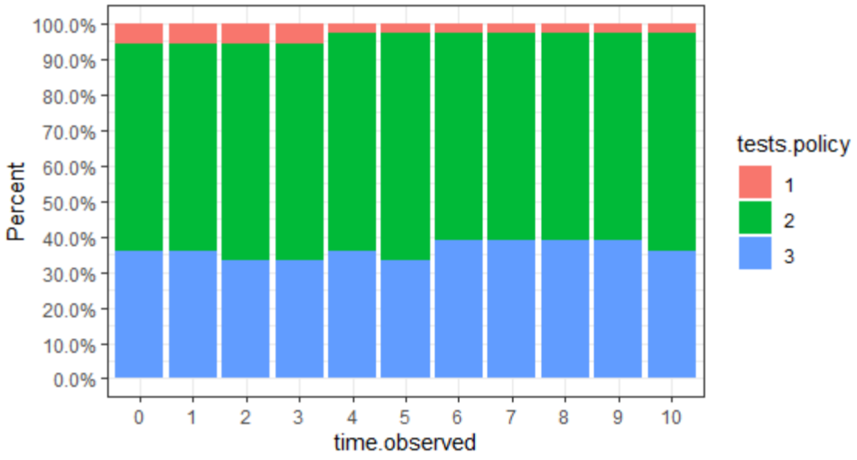


12. Distribution of the levels of intensity over time across all countries: mask wearing requirements

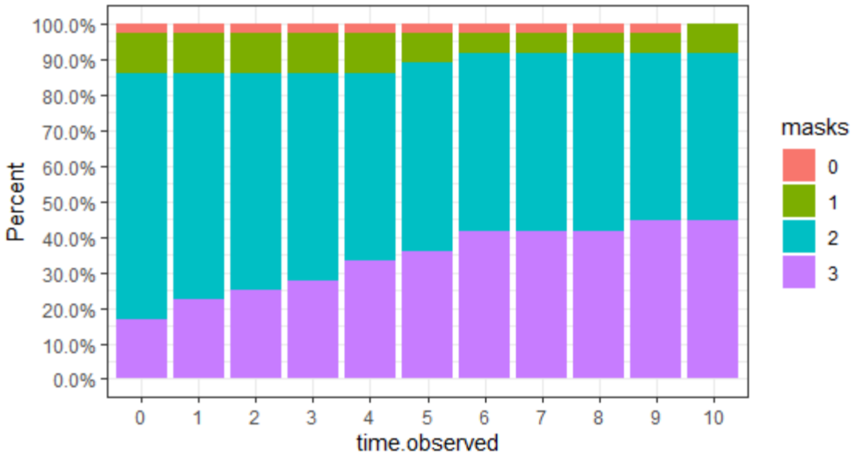

**Appendix 6. List of database variables and R script**

**1. List of database variable names used in the analysis.**

- Country = name of OECD member state
- pop2020 = total population in 2020
- urban_percent = percentage of the population living in urban areas
- SDI = sociodemographic index
- GDP_pc_PPP = GDP, purchase power parity
- GDP_health = % of GDP spent in health
- household = average household size
- palma = Palma ratio
- timely = time delay in the implementation of the first in-country social distancing NPI
- time_w_recoded = week number (0 = two weeks after first internal NPI implemented for NPI intensity / 0 = 4 weeks after first internal NPI implemented for the outcome)
- ave_daily_gr = average daily growth in the cumulative number of weekly cases (wADGR)
- strin_index = stringency index
- tests_policy = intensity of testing policy
- TT_84_pth = tests per thousand population
- contacts_policy = intensity of the contact tracing policy
- IT_1 = Intensity of international travel controls
- PI_1 = Intensity of public information campaigns
- SC_11 = Intensity of school closing requirements
- WC_11 = Intensity of workplace closing requirements
- PE_11 = Intensity of public event cancelling restrictions
- RG_11 = Intensity of restrictions on gatherings
- PT_11 = Intensity of public transport closure requirements
- SH_11 = Intensity of stay at home restrictions
- DT_11 = Intensity of internal travel restrictions
- masks = Intensity of mask-wearing policy
- temperature = temperature
- dem_index = democracy index
- mob_index = mobility composite
- cases_start = baseline number of cumulative cases

**2. R script.**

*Loading required packages*

```
pacman::p_load(INLA, INLAutils, coefINLA, brinla, buildmer, lme4, glmmTMB, TMB, lme4, lmerTest, MCMCglmm, broom, broom.helpers, DHARMa, ggplot2, plotly, ggregplot, qqplotr, epiDisplay, RColorBrewer, GGally, sjPlot, sjmisc, sjlabelled, stargazer, kableExtra, see, performance, report, parameters, modelbased, gridExtra, margins, jtools, huxtable, ggeffects, effectsize, effects, tidyverse, dplyr, devtools, tinytex, install=FALSE)
```

### 2.1. mLL model

*Calculating the probit_wADGR*

```
COVID_0207_data_2$qgr <- qnorm(COVID_0207_data_2$pgr)
```

*Setting up R database*

```
paper.update.data2 <- data.frame(

"num.id" = COVID_0207_data_2$numid,
"slope.id" = COVID_0207_data_2$slopeid,
"country" = factor(COVID_0207_data_2$Country),
"time.observed" = COVID_0207_data_2$time_w_recoded,
"qgr.response" = COVID_0207_data_2$qgr,
"adgr.response" = COVID_0207_data_2$ave_daily_gr,
"strin.index"=COVID_0207_data_2$strin_index,
"school.closing" = factor(COVID_0207_data_2$SC_11),
"work.closing" = factor(COVID_0207_data_2$WC_11),
"public.info" = factor(COVID_0207_data_2$PI_1),
"public.events" = factor(COVID_0207_data_2$PE_11),
"public.transport" = factor(COVID_0207_data_2$PT_11),
"gather.restrict" = factor(COVID_0207_data_2$RG_11),
"masks" = factor(COVID_0207_data_2$masks),
"domestic.travel" = factor(COVID_0207_data_2$DT_11),
"inter.travel" = factor(COVID_0207_data_2$IT_1),
"stay.home" = factor(COVID_0207_data_2$SH_11),
"contacts.policy" = factor(COVID_0207_data_2$contacts_policy),
"tests.policy" = factor(COVID_0207_data_2$tests_policy),
"tests.pthousand" = COVID_0207_data_2$TT_84_pth,
"temperature"=COVID_0207_data_2$temperature,
"mobility"=COVID_0207_data_2$mob_index,
"percent.urban"=COVID_0207_data_2$urban_percent,
"SDI"=COVID_0207_data_2$SDI,
"GDP.percapita"=COVID_0207_data_2$GDP_pc_PPP,
"per.GDP.health"=COVID_0207_data_2$GDP_health,
"household.size"=COVID_0207_data_2$household,
"time.delay"=COVID_0207_data_2$timely,
"initial.cases"=COVID_0207_data_2$cases_start,
"palma.ratio"=COVID_0207_data_2$palma,
"democracy.index"=COVID_0207_data_2$dem_index)
```

*Fitting univariate models (example) with lme4*

```
library(lme4)
library("lmerTest")

model_SC11 <- lmer(qgr.response~time.observed + school.closing+
                        (1+time.observed | country), data = paper.update.data2,REML=FALSE, control =
lmerControl(optimizer ="Nelder_Mead"))

summary(model_SC11)
performance(model_SC11)
```

*Fitting mLMM with lme4*

```
model_F12new <- lmer(qgr.response~time.observed +
gather.restrict+work.closing+school.closing+masks+tests.pthousand+(1+time.observed | country),
data = paper.update.data2,REML=FALSE, control = lmerControl(optimizer ="Nelder_Mead"))
```

*Running diagnostics*

```
rc_resids <- compute_redres(model_F12new)
pm_resids <- compute_redres(model_F12new, type = "pearson_mar")
sc_resids <- compute_redres(model_F12new, type = "std_cond")
resids <- data.frame(paper.update.data2$country, rc_resids, pm_resids, sc_resids)
plot_redres(model_F12new, type = "std_cond")
plot_resqq(model_F12new)
plot_ranef(model_F12new)
plot(paper.update.data2$time.observed, rc_resids,ylim=c(-1,1))
abline(h=0, col="blue")
plot(paper.update.data2$num.id, sc_resids)
abline(h=c(0,2.96,-2.96), col="blue")
```

*Fitting mLMM (Bayesian) with INLA*

```
nid <- 37

model_inla_gauss <- qgr.response ~ time.observed+work.closing + school.closing+gather.restrict +
masks+tests.pthousand + f(num.id, model="iid2d", n=2*nid) + f(slope.id, time.observed,
copy="num.id")

imod_gauss <- inla(model_inla_gauss, family="gaussian", data=paper.update.data2, verbose=FALSE)

### Regression coefficients
imod_gauss$summary.fixed[,c(1,3,5)]

plot(imod_gauss)
```

**2.2. mGLMM model (no NPI-time interactions, is easily adaptable to incorporate interactions)**

*Fitting GLMM model with glmmTMB*

```
model_final_adgr_prob <- glmmTMB(adgr.response ~ time.observed + gather.restrict + work.closing
+ school.closing + masks + tests.pthousand+(1+time.observed | country),dispformula = ~
work.closing, data = paper.update.data2, family = beta_family(link="probit"), REML = TRUE)

summary(model_final_adgr_prob)
```

*Diagnostics 1: plot of predicted versus observed values of the outcome*

```
library(modelbased)
library(dplyr)
library(ggplot2)
library(plotly)

paper.update.data2$Predicted <- estimate_response(model_final_adgr_prob)$Predicted


plot2 <- paper.update.data2 %>%
  ggplot() +
  geom_line(aes(x = log(adgr.response), y = log(adgr.response)), linetype = "dashed") +
  geom_point(aes(x = log(adgr.response) , y = log(Predicted), key=country), color = "red") +
  #geom_point(aes(x = log(adgr.response) , y = log(Predicted_2), key=country), #color = "red") +
  ylab("wADGR (predicted)") + xlab("wADGR (observed)") +
  theme_modern()
ggplotly(plot2, source = "select", tooltip = c("key") )
```

*Diagnostics 2: other diagnostics*

```
check_model(model_final_adgr_prob)
check_collinearity(model_final_adgr_prob)
plot(check_distribution(model_final_adgr_prob))

pp_check(model_final_adgr_prob, 250)

check_model(model_final_adgr_prob, check="reqq", panel = FALSE)
DHARMa::testDispersion(simulateResiduals(model_final_adgr_prob,plot=T,re.form=NULL))
DHARMa::testUniformity(model_final_adgr_prob)
DHARMa::testTemporalAutocorrelation(model_final_adgr_prob)
```

*Calculating the marginal effects*

```
# Model
model_final_adgr_probit_NO_int <- glmmTMB(adgr.response ~ time.observed + gather.restrict +
work.closing + school.closing + masks + tests.pthousand + (1+time.observed | country),dispformula
= ~ work.closing, data = paper.update.data2, family = beta_family(link="probit"), REML = TRUE)

summary(model_final_adgr_probit_NO_int)

summary(model_final_adgr_probit_NO_int)

coef_probit <- summary(model_final_adgr_probit_NO_int)$coefficients$cond[,1][-1]


AME.probit_tests.pthousand <- coef_probit[12] *
mean(dnorm(predict(model_final_adgr_probit_NO_int, type="link")))

AME.probit_time.observed <- coef_probit[1] *
mean(dnorm(predict(model_final_adgr_probit_NO_int, type="link")))

levelmasks1 <- predict(model_final_adgr_probit_NO_int,
newdata=data.frame(list("time.observed"=paper.update.data2$time.observed, "masks"="1",
"school.closing"=paper.update.data2$school.closing,
"work.closing"=paper.update.data2$work.closing,
"gather.restrict"=paper.update.data2$gather.restrict,
"tests.pthousand"=paper.update.data2$tests.pthousand, "country"=paper.update.data2$country)),
type = "response")

levelmasks0 <- predict(model_final_adgr_probit_NO_int,
newdata=data.frame(list("time.observed"=paper.update.data2$time.observed, "masks"="0",
"school.closing"=paper.update.data2$school.closing,
"work.closing"=paper.update.data2$work.closing,
"gather.restrict"=paper.update.data2$gather.restrict,
"tests.pthousand"=paper.update.data2$tests.pthousand, "country"=paper.update.data2$country)),
type = "response")

levelmasks2 <- predict(model_final_adgr_probit_NO_int,
newdata=data.frame(list("time.observed"=paper.update.data2$time.observed, "masks"="2",
"school.closing"=paper.update.data2$school.closing,
"work.closing"=paper.update.data2$work.closing,
"gather.restrict"=paper.update.data2$gather.restrict,
"tests.pthousand"=paper.update.data2$tests.pthousand, "country"=paper.update.data2$country)),
type = "response")

levelmasks3 <- predict(model_final_adgr_probit_NO_int,
newdata=data.frame(list("time.observed"=paper.update.data2$time.observed, "masks"="3",
"school.closing"=paper.update.data2$school.closing,
"work.closing"=paper.update.data2$work.closing,
"gather.restrict"=paper.update.data2$gather.restrict,
"tests.pthousand"=paper.update.data2$tests.pthousand, "country"=paper.update.data2$country)),
type = "response")
```

```
masks10 <- levelmasks1 - levelmasks0
masks20 <- levelmasks2 - levelmasks0
masks30 <- levelmasks3 - levelmasks0
AME.probit_masks1 <- mean(masks10)
AME.probit_masks2 <- mean(masks20)
AME.probit_masks3 <- mean(masks30)

levelSC0 <- predict(model_final_adgr_probit_NO_int,
newdata=data.frame(list("time.observed"=paper.update.data2$time.observed,
"masks"=paper.update.data2$masks, "school.closing"="0",
"work.closing"=paper.update.data2$work.closing,
"gather.restrict"=paper.update.data2$gather.restrict,
"tests.pthousand"=paper.update.data2$tests.pthousand, "country"=paper.update.data2$country)),
type = "response")

levelSC2 <- predict(model_final_adgr_probit_NO_int,
newdata=data.frame(list("time.observed"=paper.update.data2$time.observed,
"masks"=paper.update.data2$masks, "school.closing"="2",
"work.closing"=paper.update.data2$work.closing,
"gather.restrict"=paper.update.data2$gather.restrict,
"tests.pthousand"=paper.update.data2$tests.pthousand, "country"=paper.update.data2$country)),
type = "response")

levelSC3 <- predict(model_final_adgr_probit_NO_int,
newdata=data.frame(list("time.observed"=paper.update.data2$time.observed,
"masks"=paper.update.data2$masks, "school.closing"="3",
"work.closing"=paper.update.data2$work.closing,
"gather.restrict"=paper.update.data2$gather.restrict,
"tests.pthousand"=paper.update.data2$tests.pthousand, "country"=paper.update.data2$country)),
type = "response")

SC20 <- levelSC2 - levelSC0
SC30 <- levelSC3 - levelSC0
AME.probit_SC2 <- mean(SC20)
AME.probit_SC3 <- mean(SC30)

levelWC0 <- predict(model_final_adgr_probit_NO_int,
newdata=data.frame(list("time.observed"=paper.update.data2$time.observed,
"masks"=paper.update.data2$masks, "school.closing"=paper.update.data2$school.closing,
"work.closing"="0",
"gather.restrict"=paper.update.data2$gather.restrict,
"tests.pthousand"=paper.update.data2$tests.pthousand, "country"=paper.update.data2$country)),
type = "response")

levelWC2 <- predict(model_final_adgr_probit_NO_int,
newdata=data.frame(list("time.observed"=paper.update.data2$time.observed,
"masks"=paper.update.data2$masks, "school.closing"=paper.update.data2$school.closing,
"work.closing"="2",
```

```
"gather.restrict"=paper.update.data2$gather.restrict,
"tests.pthousand"=paper.update.data2$tests.pthousand, "country"=paper.update.data2$country)),
type = "response")

levelWC3 <- predict(model_final_adgr_probit_NO_int,
newdata=data.frame(list("time.observed"=paper.update.data2$time.observed,
"masks"=paper.update.data2$masks, "school.closing"=paper.update.data2$school.closing,
"work.closing"="3",
"gather.restrict"=paper.update.data2$gather.restrict,
"tests.pthousand"=paper.update.data2$tests.pthousand, "country"=paper.update.data2$country)),
type = "response")

WC20 <- levelWC2 - levelWC0
WC30 <- levelWC3 - levelWC0
AME.probit_WC2 <- mean(WC20)
AME.probit_WC3 <- mean(WC30)


levelGR2 <- predict(model_final_adgr_probit_NO_int,
newdata=data.frame(list("time.observed"=paper.update.data2$time.observed,
"masks"=paper.update.data2$masks, "school.closing"=paper.update.data2$school.closing,
"work.closing"=paper.update.data2$work.closing, "gather.restrict"="2",
"tests.pthousand"=paper.update.data2$tests.pthousand, "country"=paper.update.data2$country)),
type = "response")

levelGR0 <- predict(model_final_adgr_probit_NO_int,
newdata=data.frame(list("time.observed"=paper.update.data2$time.observed,
"masks"=paper.update.data2$masks, "school.closing"=paper.update.data2$school.closing,
"work.closing"=paper.update.data2$work.closing, "gather.restrict"="0",
"tests.pthousand"=paper.update.data2$tests.pthousand, "country"=paper.update.data2$country)),
type = "response")

levelGR3 <- predict(model_final_adgr_probit_NO_int,
newdata=data.frame(list("time.observed"=paper.update.data2$time.observed,
"masks"=paper.update.data2$masks, "school.closing"=paper.update.data2$school.closing,
"work.closing"=paper.update.data2$work.closing, "gather.restrict"="3",
"tests.pthousand"=paper.update.data2$tests.pthousand, "country"=paper.update.data2$country)),
type = "response")

levelGR4 <- predict(model_final_adgr_probit_NO_int,
newdata=data.frame(list("time.observed"=paper.update.data2$time.observed,
"masks"=paper.update.data2$masks, "school.closing"=paper.update.data2$school.closing,
"work.closing"=paper.update.data2$work.closing, "gather.restrict"="4",
"tests.pthousand"=paper.update.data2$tests.pthousand, "country"=paper.update.data2$country)),
type = "response")

GR20 <- levelGR2 - levelGR0
GR30 <- levelGR3 - levelGR0
GR40 <- levelGR4 - levelGR0
AME.probit_GR2 <- mean(GR20)
AME.probit_GR3 <- mean(GR30)
```

```
AME.probit_GR4 <- mean(GR40)

AME.probit.noint <- data.frame("time.observed" = AME.probit_time.observed, "masks1" =
AME.probit_masks1, "masks2" = AME.probit_masks2, "masks3" = AME.probit_masks3,
"school.closing2" = AME.probit_SC2, "school.closing3" = AME.probit_SC3, "work.closing2" =
AME.probit_WC2, "work.closing3" =  AME.probit_WC3, "gather.restrict2" = AME.probit_GR2,
"gather.restrict3" = AME.probit_GR3, "gather.restrict4" = AME.probit_GR4, "tests.pthousand" =
AME.probit_tests.pthousand)

rownames(AME.probit.noint) <- "AME.noint"
t(AME.probit.noint)
```

REFERENCES:

1. Leffler CT, Ing E, Lykins JD, Hogan MC, McKeown CA, Grzybowski A. Association of Country-wide Coronavirus Mortality with Demographics, Testing, Lockdowns, and Public Wearing of Masks. The American Journal of Tropical Medicine and Hygiene. 2020;103(6):2400-11. doi:10.4269/ajtmh.20-1015.

2. World Health Organisation. Tracking Public Health and Social Measures A Global Dataset. 2020. https://www.who.int/emergencies/diseases/novel-coronavirus-2019/phsm.
